# Wildlife as a reservoir of OXA-48-like carbapenemase-producing Enterobacterales

**DOI:** 10.1128/spectrum.00432-26

**Published:** 2026-07-21

**Authors:** Imene Mehidi, Alima Sait-Gharout, Abdelazize Franck Bougaham, Hocine Gougam, Bogdan I. Iorga, Riadh Moulai, Laurent Dortet, Rémy A. Bonnin, Delphine Girlich, Mourad Ahmim, Saoussen Oueslati, Thierry Naas

**Affiliations:** 1 Laboratoire d’Ecologie Microbienne, FSNV, Université de Bejaia, Bejaia, Algérie; 2 Team ReSIST INSERM U1184, School of Medicine Université Paris-Saclay, Health and Therapeutic Innovation object (Healthi), Le Kremlin-Bicêtre, France; 3 Inserm, CEA, Center for Immunology of Viral, Auto-immune, Hematological and Bacterial diseases (IMVA-HB/IDMIT/UMRS1184), Université Paris-Saclay 568414 , Le Kremlin-Bicêtre, France; 4 Laboratoire d’Écologie et Environnement, FSNV, Université de Bejaia, Bejaia, Algérie; 5 Institut de Chimie des Substances Naturelles, CNRS UPR 2301, Université Paris-Saclay, Gif-sur-Yvette, France; 6 Laboratoire de Zoologie Appliquée et d’Ecophysiologie Animale, FSNV, Université de Bejaia, Bejaia, Algérie; 7 French National Reference Center for Antibiotic Resistance: Carbapenemase-Producing Enterobacterales, Le Kremlin-Bicêtre, France; 8 SEPSIS Comprehensive Center—IHU SEPSIS, Le Kremlin-Bicêtre, France; JMI Laboratories, North Liberty, Iowa, USA

**Keywords:** OXA-48-like, carbapenemases, captive animals, wild animals

## Abstract

Carbapenemase-producing Enterobacterales (CPEs) have globally emerged and spread beyond human compartments. However, data in wild animals, especially from low- and middle-income countries, such as Algeria, are still very scarce. Here, we investigated CPEs recovered from feces samples collected between October 2021 and June 2023 from wild terrestrial and aquatic mammals, wild migratory/nesters/sedentary birds, and zoo animals, including their environment (water, food, and fecal samples of animal care workers) distributed over six Algerian provinces. Carbapenem-resistant Enterobacterales were characterized using MALDI-TOF-MS, Carba NP, immunochromatographic assay NG-Test CARBA 5, antimicrobial susceptibility testing, and whole-genome sequencing. Thirty CPEs were identified out of the 1,899 samples collected (1.6%). The carriage rate was higher in captive animals (3.2%) than in wild animals (1.2%). Twenty-six produced OXA-48, three OXA-244, and one OXA-181, along with CTX-M-15 ESBL. Clonal expansion of *Enterobacter hormaechei hoffmannii* ST145 and *Klebsiella pneumoniae* ST13 was evidenced. Plasmid analysis confirmed that 24/30 isolates harbored a transferable 62 kb IncL pOXA-48 plasmid. Five/six *E. coli* isolates belonged to high-risk clones with chromosome-mediated *bla*_OXA-244_ gene in three isolates, *bla*_OXA-48_ in two isolates, and *bla*_OXA-181_ gene encoded on an IncFII-ColKP3 hybrid plasmid in one isolate. This study showed widespread dissemination of OXA-48-like producing Enterobacterales in free and captive wild animals, largely driven by epidemic plasmids and clones. It underscores the role of wild animals as a reservoir of CPEs, particularly species living close to humans, such as gulls and pigeons, and occasionally food-producing animals, increasing the risk of bidirectional dissemination between animal, environmental, and human sectors.

**IMPORTANCE:**

The global rise of carbapenemase-producing Enterobacterales (CPEs) harboring *bla*_OXA-48-like_ has been increasingly documented in clinical settings. However, their emergence and transmission in wild and captive animals are less documented. This study provides a high-resolution genomic characterization of CPEs isolated from the feces of wild animals, especially migratory birds, and from captive wild animals, to evaluate the potential risk of dissemination through these animals. Whole-genome sequencing data, genetic investigations, and antimicrobial susceptibility results highlighted the spread of multidrug-resistant CPEs in both animals and humans. The widespread detection of *bla*_OXA-48_ across multiple niches suggests sustained circulation beyond hospital settings in Algeria. Human-associated lineages, such as *E. coli* ST131, ST38, and ST540, were identified with a clear link with humans. This study demonstrates carriage of CPEs in multiple bird species living in areas commonly inhabited by humans and provides further evidence for an effective dissemination of resistance in wildlife, facilitated by feeding habits.

## INTRODUCTION

The emergence of multi-drug resistant (MDR) bacteria in both humans and animals is becoming a major public health concern, particularly with carbapenem-resistant Enterobacterales (1, 2). The main resistance mechanism in Enterobacterales is related to the production of carbapenem-hydrolyzing enzymes, carbapenemases (2). OXA-48, initially described in a *Klebsiella pneumoniae* strain in Turkey in 2004, has become the most common carbapenemase in countries around the Mediterranean rim, including Algeria (3–5). Along with its spread, more than 40 variants of OXA-48 have been reported, including OXA-244, which exhibits lower hydrolytic activity against carbapenems and temocillin compared to OXA-48, making its detection difficult and allowing silent spread (6–10).

Carbapenemase-producing Enterobacterales (CPE) have become a major health issue, requiring a “One Health” integrated approach (11). International travel is an important vector for the importation of CPE into low-prevalence countries (2, 4). Many studies have addressed the occurrence of antibiotic resistance genes in livestock or companion animals, but few have investigated the potential role of wildlife in the spread of CPEs. Wildlife has been suggested to contribute to the maintenance and transmission of pathogens with a gradient in the frequency and diversity of antibiotic-resistant bacteria from the most human-impacted areas, which are the most affected, to the most preserved areas (12). In Algeria, studies have reported the presence of CPE in wild animals, notably OXA-48 in wild boars, Barbary macaques, bat guano, and pigeons (5, 13–15).

Zoos, due to their ability to bring exotic animals into close contact with humans, may facilitate the spread of MDR bacteria (16–18). Due to the increasing popularity of zoos and their ability to bring exotic animals into proximity to humans, zoos may facilitate the spread of MDR bacteria between other animals, humans, and the environment. In addition, zoos may also facilitate the transfer of resistant bacteria and/or resistance genes to other animals and the environment, as animals are often exchanged as part of breeding programs and due to the reintroduction of zoo animals and/or their offspring into the wild. A few studies have reported the presence of ESBL- and/or AmpC-producing Enterobacterales in zoo animals, with prevalence rates ranging from 12% to 32%, such as in Israel 12% (*n* = 228), the Czech Republic 31% (*n* = 160), and China 32% (*n* = 206) (19–22).

Here, we determined the prevalence of CPEs isolated from free and captive wild animals in Algeria, their molecular mechanisms sustaining carbapenem resistance, and their geographic distribution. This is the first study on CPEs isolated from wild zoo animals in Africa.

## MATERIALS AND METHODS

### Sample collection

**Fig 1:**
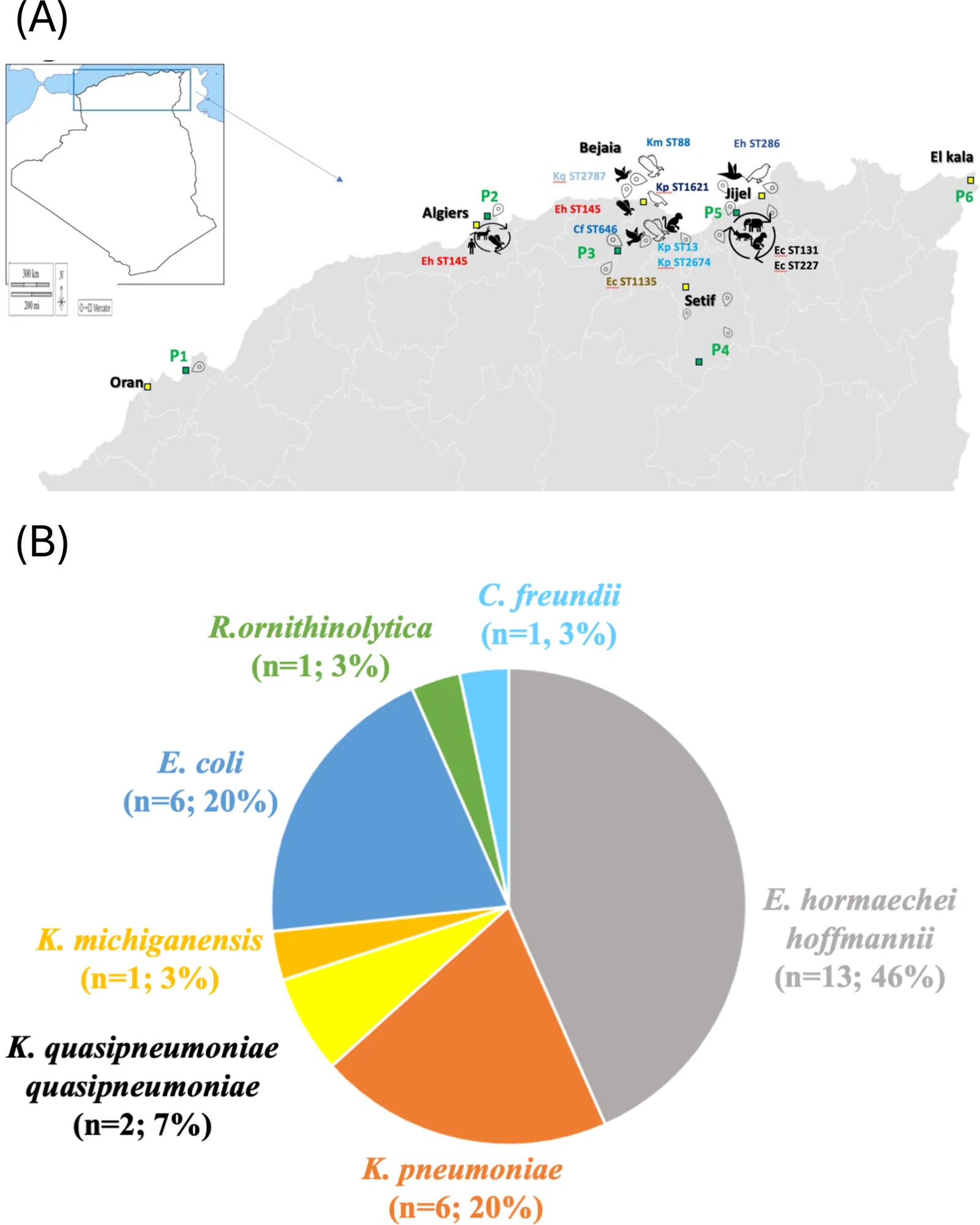
Sampling sites and CPEs. (**A**) Sampling locations and distribution of the OXA-48-like producing Enterobacterales clones circulating among wild and captive animals in Algeria, and (**B**) CPE species identified. P: zoological park: P1, Arche de Noe in Oran; P2, El Hamma du Jardin d'essai in Algiers; P3, Keffous in Bejaia; P4, Hadj Aissa in Setif; P5, El Aouana Boudjeblida in Jijel; P6, Brabtia in El Kala. *Eh*, *Enterobacter hormaechei hoffmannii*; *Kp*, *Klebsiella pneumoniae; Kq*, *Klebsiella quasipneumoniae quasipneumoniae*; *Km*, *Klebsiella michiganensis*; *Ec*, *Escherichia coli*; *Cf*, *Citrobacter freundii.*

Samples were collected from diverse ecological niches across six Algerian provinces between October 2021 and June 2023, including free-living wild animals (*n* = 1,521) and captive animals in six zoos (*n* = 378; Fig. 1A and Table 1).

**TABLE 1:** Numbers of samples collected from the different ecological niches in Algeria and distribution of OXA-48-producing Enterobacterales

Sampling locations	Samples collected	OXA-48-producing Enterobacterales (%)
Wildlife		
Bejaia	1,404	16 (1.1%)
Setif	48	0
Jijel	69	2 (2.9%)
Total wildlife	1,521	18 (1.2%)
Captive wildlife (zoological parks)		
P1: Arche de Noe in Oran	37	0
P2: El Hamma du Jardin d'essai in Algiers		
Animals	120	8 (6.6%)
Zoo workers	9	1 (11%)
P3: Keffous in Bejaia		
Animals	47	0
Zoo workers	4	0
P4: Hadj Aissa in Setif	54	0
P5: El Aouana Boudjeblida in Jijel	62	3 (4.8%)
P6: Brabtia in El Kala	45	0
Total captive wildlife	365	11 (3.0%)
Total zoo workers	13	1 (7.7%)
Total	1,899	30 (1.6%)

Free-living animal samples comprised fresh feces, fish, and sea urchin droppings and were mostly from migratory, sedentary, and breeding birds, as well as land and marine species (Table S1). All fecal samples from free-ranging wildlife were collected under a strictly defined protocol to ensure reliable species attribution. Feces from terrestrial mammals and bird droppings were collected fresh, either immediately after direct observation of defecation or under conditions allowing unambiguous species identification (e.g., isolated presence of the individual, distinctive morphological characteristics, and ecological context). Sampling sites were selected based on the known habitats of the target species. Fieldwork was conducted with the support of ornithologists—specialists in avian ecology, behavior, and systematics—who contributed to species identification, site selection, and the discrimination of droppings using morphological, ecological, and contextual criteria. Samples from bats consisted of guano collected from previously identified and characterized natural roosting sites (caves/cavities). Species attribution was based on prior knowledge of the colonies inhabiting these sites, under the supervision of a specialist in chiropterans. For marine animals (mainly sea bream and sea urchins), samples were obtained directly from each individual through dissection and collection of the gastrointestinal tract under controlled conditions, thereby preventing cross-contamination and sample traceability at the individual level.

Captive animal samples included bird (*n* = 136), mammal (*n* = 195), and reptile droppings (*n* = 21), along with water (*n* = 7), food (*n* = 6), and zoo worker stool samples (*n* = 13; Table S2). In all zoos, antimicrobials are used only for therapeutic purposes under veterinary supervision. Fecal samples were collected early in the morning using sterile swabs and containers, avoiding duplicates, with sampling limited to the number of animals per pen (maximum five samples/pen). The number of samples was limited to the number of animals per pen, with a maximum of five samples for pens housing five or more animals. For crocodiles and other tank-housed animals, three 500 mL water samples were taken before disinfection. Rectal swabs were self-collected by healthcare staff using Amies-based swabs (Oxoid, Algiers, Algeria).

### Isolation and identification of carbapenem-resistant bacteria

Samples were transported in an isothermal box at 4°C within 6 h of collection and processed within 24 h at the Microbial Ecology laboratory of the University of Bejaia. Samples were enriched in trypticase soy broth (TSB, Fluka, St. Louis, USA) supplemented with ertapenem (0.5 mg/L) and vancomycin (32 mg/L) and incubated for 18 h at 37°C. For food products, 25 g of each food product from the zoos was cultured in 225 mL of TSB supplemented with the same antibiotics as described above and incubated for 18 h at 37°C. After incubation, an aliquot of 200 µL was plated onto MacConkey agar (Fluka) containing 0.5 mg/L ertapenem and incubated for 18–24 h at 37°C. For water and wastewater samples, the isolation procedure involved inoculating 200 µL aliquots of samples diluted 1:10 on MacConkey agar containing 0.5 mg/L ertapenem. The resulting colonies isolated from the various screening agars were then identified by MALDI-TOF (Maldi Biotyper, Bruker, Hamburg, Germany).

### Susceptibility testing

Initial susceptibility testing was done using the Kirby-Bauer disc diffusion method (23). Additionally, MIC values for CPEs were determined using broth microdilution assay (Seq2Diag plates 1, 2, and 3, Sensititre, ThermoFisher, Grenoble, France) for 47 antibiotics, including novel β-lactams and non-β-lactam antibiotics of clinical and veterinary interest (Table 2). Susceptibility patterns were interpreted according to the EUCAST guidelines (https://www.eucast.org/clinical_breakpoints) for human antibiotics and veterinary guidelines for animal-restricted antibiotics (https://www.sfm-microbiologie.org/wp-content/uploads/2021/12/CASFM_VET2021.pdf).

**TABLE 2:** Resistome of the 30 CPE isolates and plasmid incompatibility types identified

									β-lactams							Aminosides							Quinolones							Various							Plasmids						
Isolate #	WGS Id^a^	Date of isolation (day/mo/yr)	Region	Niche	MLST	NG test carba 5	Carba NP test	(C/P)^b^	OXA-48-like	OXA-181	CTX-M-15	DHA-1	CMY-	ACT-	TEM-	SHV-	OXY-1-4	ORN-1	OKP-A-	AadA-	Aph(3')-Ia	Aac(3)-IId	Qnr-like	OqxA	OqxB	Sul1	Mph(A)	Erm(B)	Tet-	CmlA4	DfrA-	fosA-like	IncFIA	IncFIB	IncFIII	IncHI1B	IncL	IncR	IncN	IncX-	IncY	repB	Col-like
O97H1	Eh	03/07/22	Jijel	Dipper (*Cinclus cinclus*)	286	OXA	+	P	48^c^					23^c^																													
O97G10	Eh	01/07/22	Jijel	White Wagtail (*Motacilla cinerea*)	286	OXA	+	P	48					23																													
O98A2	Eh	01/03/22	Zoo, Algier	Red deer (*Dama dama*)	145	OXA	+	P	48					90	40																								2				
O97I8	Eh	01/03/22	Zoo, Algier	Amazon (*Amazona ochrocephala*) Cockatoo (*Cacatua alba*)	145	OXA	+	P	48					90	40																								2				
O98A6	Eh	01/03/22	Zoo, Algier	Silver pheasant (*Lophura nycthemera*)	145	OXA	+	P	48					90	40																								2				
O98A7	Eh	01/03/22	Zoo, Algier	Gabonese gray (*Psittacus erithacus*)	145	OXA	+	P	48					90	40																								2				
O97I9	Eh	01/03/22	Zoo, Algier	Striped Hyena (*Hyaena hyaena*)	145	OXA	+	P	48					90	40																								2				
O98A8	Eh	01/03/22	Zoo, Algier	Cuvier gazelle (*Gazella cuvieri*)	145	OXA	+	P	48					90	40																								2				
O97I10	Eh	01/03/22	Zoo, Algier	Callopsite parakeet (*Nymphicus hollandicus*)	145	OXA	+	P	48					90	40																								2				
O97J1	Eh	01/03/22	Zoo, Algier	Human Screening	145	OXA	+	P	48					90	40																								2				
O97J2	Eh	01/03/22	Zoo, Algier	Yellow-crested Cockatoo (*Cacatua galerita*)	145	OXA	+	P	48					90	40																								2				
O97H7	Eh	27/03/22	Bejaia	Cattle Egret (*Bubulcus ibis*)	145	OXA	+	P	48					90	40																								2				
O97I2	Eh	08/03/22	Bejaia	Black-headed gull (*Chroicocephalus ridibundus*)	145	OXA	+	P	48					90	40																								2				
O97H4	Kp	08/03/22	Bejaia	Yellow-legged Gull (*Larus michahellis*)	13	OXA	+	P	48							1																											
O97J10	Kp	08/03/22	Bejaia	Yellow-legged Gull (*Larus michahellis*)	13	OXA	+	P	48							1																											
098A1	Kp	08/03/22	Bejaia	Yellow-legged Gull (*Larus michahellis*)	13	OXA	+	P	48							1																											
O98A4	Kp	08/03/22	Bejaia	Black-headed gull (*Chroicocephalus ridibundus*)	13	OXA	+	P	48							1																											
O97H5	Kp	12/07/22	Bejaia	House swallow (*Delichon urbica*)	1,621	OXA	+	P	48							1													D														
O97I1	Kp	09/10/21	Bejaia	Magot monkey (*Macaca sylvanus*)	2,674	OXA	+	P	48							172																											
O97J9	Kq	09/10/21	Bejaia	Yellow-legged Gull (*Larus michahellis*)	2,787	OXA	+	P	48										4																								
O97I4	Kq	08/03/22	Bejaia	Black-headed gull (*Chroicocephalus ridibundus*)	–	OXA	+	P	48										7																								
O98A5	Km	08/03/22	Bejaia	Black-headed gull (*Chroicocephalus ridibundus*)	88	OXA	+	P	48											2b											15								1				
O97H9	Ec	28/03/22	Jijel	Capuchin (*Cebus capucinus*)	131	OXA	−	C	244											5											17												
O97H10	Ec	28/03/22	Jijel	Elephant (*Loxodonta africana*)	131	OXA	−	C	244																																		
O97I5	Ec	08/03/22	Bejaia	Black-headed gull (*Chroicocephalus ridibundus*)	1,135	OXA	−	C	244				2		1					2											12									4			
O97H6	Ec	30/01/22	Bejaia	Cattle Egret (*Bubulcus ibis*)	38	OXA	+	C	48				2		1					5											17												
O97H8	Ec	28/03/22	Jijel	Fennec (*Vulpes zerda*)	227	OXA	+	C	48																				A														
O100E1	Ec	08/03/22	Bejaia	Black-headed gull (*Chroicocephalus ridibundus*)	540	OXA	−	P		181					1								S1						B											3			
O97J6	Ro	08/03/22	Bejaia	Black-headed gull (*Chroicocephalus ridibundus*)	–	OXA	+	P	48																																		
O97I7	Cf	08/03/22	Bejaia	Black-headed gull (*Chroicocephalus ridibundus*)	646	OXA	+	P	48				82										B10						E														

^a^ *Eh*,* E. hormaechei hoffmannii*;* Kp*,* K. pneumoniae*;* Kq*,* K. quasipneumoniae quasipneumoniae*;* Km*,* K. michiganensis*;* Ec*,* E. coli*;* Ro*,* R. ornithinolytica*;* Cf*,* C. freundii.*

^b^ C, chromosomal insertion; P, plasmidic position of bla_OXA-48-like._

^c^ Gray boxes indicate presence, and a number in the boxes indicates the allelic number of a given gene.

### Phenotypic detection of carbapenemases

The home-made Carba NP test and the lateral flow immunoassay NG-Test CARBA 5 (NG Biotech, Guipry, France) for the detection of the five main carbapenemases (KPC-, NDM-, VIM-, IMP-, and OXA-48-like) were performed as previously described on bacteria with reduced susceptibility to carbapenems following EUCAST recommendation (ertapenem diameter <25 mm; https://www.eucast.org/clinical_breakpoints) (24, 25).

### Whole-genome sequencing

Genomic DNA was extracted using the PureLink genomic DNA Mini Kit (Thermofisher) following the manufacturer’s instructions. Dual-indexed sequencing libraries were constructed using the NEBNext library preparation kit and the Multiplex Oligos for Illumina (NEB, Boston, MA, USA). The libraries were sequenced on an Illumina Next 500 (2 × 150 bp). Genome assembly was performed using CLC Genomics Workbench v12 (Qiagen, Les Ulis, France). Online bioinformatics tools available at the Center for Genomic Epidemiology (https://www.genomicepidemiology.org/services/) were used to characterize the isolates’ resistome, MLST, genetic relatedness, and plasmid replicons. Two OXA-48-producing *E. coli* isolates were further sequenced using long-read technology on a Minion sequencer (Nanopore, Cambridge, UK). Hybrid assemblies were done using CLC Genomics Workbench v12 (Qiagen). In addition, Illumina reads were mapped to the prototypical pOXA-48 plasmid (26) or to *bla*_OXA-181_ carrying plasmid of 51,479 kb plasmid (accession number CP023897), using CLC Genomics Workbench v12 (Qiagen). For the OXA-244 producers, PCR approaches were used to precisely map the insertion into the chromosome of *E. coli* (27). We have set the single-nucleotide polymorphism (SNP) thresholds to define epidemiological relatedness to <50 SNPs, as this value has been used in several studies as a conservative cutoff to exclude recent epidemiological linkage (28). Whole-genome sequences (WGSs) have been deposited at DDBJ/ENA/GenBank under bioproject PRJNA1242567. Specific accession numbers are listed in Table S3.

#### Plasmid characterization

The plasmids were extracted using the Kieser method and analyzed as previously described (29, 30). Extracted plasmids were electroporated into *E. coli* TOP10 (Invitrogen, Saint-Aubin, France) and transformants selected on TSA agar plates supplemented with either temocillin 100 µg/mL or ticarcillin 50 µg/mL. Transformants were confirmed by antimicrobial susceptibility and PCR detection of the *bla*_OXA-48_ gene (31). For isolates for which no transformants could be obtained, mating-out assays were performed using azide-resistant *E. coli* J53 as the recipient (31). Plasmids of *ca*. 154, 66, 48, and 7 kb of *E. coli* NCTC 50192 were used as plasmid size markers (32).

## RESULTS

### CPEs identified

A total of 1,899 samples (1,521 from wild animals, 365 from captive animals, and 13 from animal care workers) were collected between October 2021 and June 2023 (Fig. 1A and Table 1). Bacterial growth occurred on ertapenem-supplemented plates for 10.74% of the total samples (204/1,899), and for 33.8% (128/378) from captive wild animals and 5% (76/1,521) from free-roaming wild animals, but only 1.6% (30/1,899) of the samples grew with CPEs. Carba NP test was positive for 26/30 isolates, while NG-Test CARBA-5 gave 30 positive results for OXA-48-like enzymes. These 30 CPEs corresponded to *Enterobacter hormaechei hoffmannii* (*Eh*; *n* = 13), *K. pneumoniae* (*Kp*; *n* = 6), *E. coli* (*Ec*; *n* = 6), *Klebsiella quasipneumoniae quasipneumoniae* (*Kq*; *n* = 2), *Klebsiella michiganensis* (*Km*; *n* = 1), *Raoutella ornithinolytica* (*Ro*; *n* = 1), and *Citrobacter freundii* (*Cf*; *n* = 1; Fig. 1B). One CPE originated from the rectal swab sample of a zoo animal care worker (*n* = 1/13; 7.7%), 11 from captive animals (*n* = 11/365; 3.0%), and 18 (18/1,521; 1.2%) from free-roaming wild animals. (Tables S1 and S2).

### Resistome

WGS of the 30 CPEs revealed 26 OXA-48-, 3 OXA-244-, and one OXA-181-producers, the latter corresponded to MDR-*Ec* isolates co-expressing *bla*_CTX-M-15_ gene (Table 2). Host-specific and chromosome-borne β-lactamase genes such as *bla*_SHV_ were identified in all *Kp* isolates, *bla*_OKP-A-7_ gene in *Kq*, *bla*_ORN-1_ in *Ro*, *bla*_OXY-1-4_ in *Km*, *bla*_ACT-_ in all *Eh* isolates, and *bla*_CMY-82_ in *Cf* isolate, while plasmid-encoded cephalosporinases *bla*_CMY-2_ and *bla*_DHA-1_ genes were found in two *Ec* and in *Km,* respectively. Complete list of acquired resistance genes is indicated in Table 3.

**TABLE 3:** Susceptibility testing for 18 representative CPEs

	EUCAST breakpoints (mg/L)^*a*^		MIC (µg/ml)^*b*^																	
Substrate	S≤	R>	Eh O97H1 ST286	Eh 097G10 ST286	Eh O97J1 ST145	Eh O97H7 ST145	Eh 097I2 ST145	Kp 097H4 ST13	Kp 098A4 ST13	Kp 097H5 ST1621	Kp 097I1 ST2574	Kq 097J9 ST2787	Km 098A5 ST88	Ec 097H9 ST131	Ec O97H10 ST131	Ec 097I5 ST1135	Ec 097H8 ST227	Ec O100E1 ST540	Ro 097I6 -	Cf 097I7 ST646
Amoxicillin	8	8	>32	>32	>32	>32	>32	>32	>32	>32	>32	>32	>32	>32	>32	>32	>32	>32	>32	>32
Amoxicillin/clavulanic acid	8	8	>128	>128	>128	>128	>128	>128	>128	>128	>128	>128	>128	>128	>128	>128	>128	>128	>128	>128
Ticarcillin	–^*i*^	–	>32	>32	>32	>32	>32	>32	>32	>32	>32	>32	>32	>32	>32	>32	>32	>32	>32	>32
Temocillin	0.001	16	>512	>512	>512	>512	>512	512	512	512	512	512	512	128	256	256	512	512	256	512
Piperacilline	8	8	>32	>32	>32	>32	>32	>32	>32	>32	>32	>32	>32	>32	>32	>32	>32	>32	>32	>32
Piperacilline/tazobactam	8	8	>32	>32	>32	>32	>32	>32	>32	>32	>32	>32	>32	>32	>32	>32	>32	>32	>32	>32
Mecillinam	8	8	>16	>16	16	8	16	16	>16	>16	>16	>16	16	>16	>16	>16	4	8	16	16
Cefalexine	16	16	128	64	32	>128	16	8	8	16	8	16	16	>128	>128	>128	8	128	8	128
Cefoxitin	–	–	>32	>32	16	>32	8	≤4	≤4	8	8	8	≤4	8	8	>32	≤4	8	≤4	>32
Cefotaxime	1	2	4	4	2	>8	2	1	1	1	1	1	1	>8	>8	>8	≤0.5	8	≤0.5	1
Ceftazidime	1	4	0.5	0.5	0.5	16	0.5	≤0.25	0.5	1	0.5	0.5	0.5	16	16	32	≤0.25	16	≤0.25	0.5
Ceftazidime/avibactam	8	8	0.5	0.5	≤0.25	≤0.25	0.5	≤0.25	≤0.25	0.5	0.5	0.5	≤0.25	≤0.25	≤0.25	≤0.25	≤0.25	≤0.25	≤0.25	≤0.25
Cefepime	1	4	≤0.5	1	≤0.5	≤0.5	≤0.5	≤0.5	1	≤0.5	≤0.5	≤0.5	≤0.5	16	16	16	≤0.5	8	≤0.5	≤0.5
Cefiderocol	2	2	0.5	0.5	≤0.03	≤0.03	0.12	≤0.03	≤0.03	0.5	≤0.03	≤0.03	≤0.03	0.5	0.5	0.25	0.06	0.25	≤0.03	≤0.03
Ceftaroline	0.5	0.5	>2	>2	>2	>2	>2	>2	>2	>2	>2	>2	>2	>2	>2	>2	>2	2	>2	>2
Ceftiofur^*a*^	2	4	8	4	4	8	4	2	2	4	4	4	4	>32	>32	>32	≤1	32	≤1	2
Ceftobiprole	0.25	0.25	>1	>1	>1	>1	>1	>1	>1	>1	>1	>1	>1	>1	>1	>1	>1	>1	>1	>1
Aztreonam	1	4	0.12	0.12	0.12	4	≤0.06	≤0.06	≤0.06	0.12	0.12	0.12	≤0.06	>16	>16	>16	0.12	16	≤0.06	0.12
Aztreonam/avibactam	4	4	0.12	0.12	0.12	0.12	0.06	≤0.03	0.06	0.06	0.06	0.06	0.5	0.12	≤0.03	0.06	0.06	0.06	0.06	0.06
Ceftolozane/tazobactam	2	2	2	2	1	4	1	1	2	2	2	2	2	8	16	>16	0.5	2	0.5	0.5
Ertapenem	0.5	0.5	8	>8	4	4	4	2	2	2	2	4	4	1	2	2	0.05	1	2	0.5
Imipenem	2	4	4	4	1	0.5	1	2	2	2	2	2	1	0.5	1	0.5	0.5	0.5	2	2
Imipenem/relebactam	2	2	4	4	1	1	1	1	1	1	2	2	1	0.25	0.5	0.25	0.5	0.5	1	2
Meropenem	2	8	4	4	1	0.5	1	1	2	2	1	2	1	0.25	0.5	0.5	0.25	0.25	1	1
Meropenem/vaborbactam	8	8	4	2	1	0.5	1	0.5	1	1	1	1	1	0.12	0.5	0.5	0.12	0.25	1	0.5
Norfloxacin	0.5	0.5	0.12	0.25	0.12	0.5	0.12	0.25	0.25	0.25	0.25	0.25	0.25	>2	>2	1	0.12	2	≤0.06	≤0.06
Ciprofloxacin	0.06	0.06	≤0.06	≤0.06	≤0.06	0.12	≤0.06	≤0.06	≤0.06	≤0.06	≤0.06	≤0.06	≤0.06	2	2	0.25	≤0.06	2	≤0.06	≤0.06
Deflafloxacin	0.125	0.125	0.06	0.03	0.03	0.03	0.06	0.06	0.03	0.03	0.06	0.06	0.03	0.5	0.5	0.12	0.03	2	≤0.015	0.03
Pradofloxacin^*c*^	2	2	≤0.12	≤0.12	≤0.12	≤0.12	≤0.12	≤0.12	≤0.12	≤0.12	≤0.12	≤0.12	≤0.12	0.5	0.5	≤0.12	≤0.12	2	≤0.12	≤0.12
Levofloxacin	0.5	1	≤0.12	≤0.12	≤0.12	≤0.12	≤0.12	≤0.12	≤0.12	≤0.12	≤0.12	≤0.12	≤0.12	1	1	0.5	≤0.12	2	≤0.12	≤0.12
Florfenicol^*c*^	8	16	8	4	8	8	8	4	4	8	8	4	4	16	8	16	8	8	≤2	8
Chloramphenicol^*d*^	16	16	8	≤4	8	16	8	≤4	≤4	≤4	8	≤4	≤4	16	16	16	8	8	≤4	8
Tetracycline^*e*^	4	4	4	4	4	≤2	4	≤2	≤2	>16	≤2	4	≤2	≤2	≤2	>16	>16	16	≤2	>16
Tigecycline^*f*^	0.5	0.5	0.5	0.5	0.5	≤0.25	2	0.5	0.5	2	0.5	0.5	0.5	≤0.25	≤0.25	≤0.25	0.5	0.5	0.5	0.5
Eravacycline	0.5	0.5	0.5	0.5	0.5	0.12	0.5	0.25	0.25	0.25	0.25	0.25	0.25	0.12	0.12	0.12	0.25	0.25	0.12	0.5
Tobramycin	2	2	≤1	≤1	≤1	4	≤1	≤1	≤1	≤1	≤1	≤1	≤1	≤1	≤1	≤1	≤1	≤1	≤1	≤1
Gentamicin	2	2	≤1	≤1	≤1	>8	≤1	≤1	≤1	≤1	>8	≤1	≤1	≤1	≤1	≤1	≤1	≤1	≤1	≤1
Amikacin	8	8	≤4	≤4	≤4	≤4	≤4	≤4	≤4	≤4	≤4	≤4	≤4	≤4	≤4	≤4	≤4	≤4	≤4	≤4
Apramycine^*c*^	16	16	16	4	2	4	2	2	2	2	2	2	2	4	4	4	16	4	1	4
Neomycine^*c*^	8	16	≤4	≤4	≤4	≤4	≤4	≤4	≤4	≤4	≤4	≤ 4	≤4	≤4	≤4	≤4	≤4	≤4	≤4	≤4
Streptomycine^*c*^	8	16	≤4	8	≤4	16	≤4	≤4	≤4	≤4	>32	≤4	≤4	16	16	16	8	8	≤4	16
Sulfamethoxazole^*c*^	64	256	≤32	≤32	≤32	>512	≤32	≤32	≤32	≤32	≤32	≤32	≤32	>512	>512	>512	≤32	≤32	≤32	≤32
Trimethoprim	4	4	≤1	≤1	≤1	>8	≤1	≤1	≤1	≤1	≤1	≤1	≤1	>8	>8	>8	≤1	≤1	≤1	≤1
Trimethoprim-sulfamethoxazole	2	4	≤1	≤1	≤1	>8	≤1	≤1	≤1	≤1	≤1	≤1	≤1	>8	>8	>8	≤1	≤1	≤1	≤1
Colistin	2	2	≤0.5	≤0.5	≤0.5	1	1	≤0.5	≤0.5	1	≤0.5	≤0.5	≤0.5	≤0.5	≤0.5	≤0.5	≤0.5	≤0.5	≤0.5	≤0.5
Nitrofurantoin^*g*^	64	64	64	≤32	≤32	≤32	≤32	≤32	≤32	64	64	≤32	64	≤32	≤32	≤32	≤32	≤32	≤32	≤32
FOS G6P^*h*^	8	8	16	16	16	≤4	16	16	>32	32	16	32	>32	≤4	>32	≤4	≤4	≤4	32	8

^*a*^ EUCAST breakpoints are used, unless indicated.

^*b*^ MIC interpretation according to EUCAST guidelines, unless specified: gray shading: resistant; underlined values: susceptible with high dosing; white: susceptible. Eh, *E. hormaechei hoffmannii*; Kp, *K. pneumoniae*; Kq, *K. quasipneumoniae quasipneumoniae*; Km, *K. michiganensis*; Ec, *E. coli*; Ro, *R. ornithinolytica*; Cf, *C. freundii.*

^*c*^ Veterinary breakpoints were used (https://www.sfm-microbiologie.org/wp-content/uploads/2023/06/CASFM_VET2023.pdf?).

^*d*^ Screening cut-off values were used to distinguish wild-type isolates from isolates with acquired resistance.

^*e*^ Tetracycline MIC ≤ 4 mg/L for wild-type isolates.

^*f*^ Breakpoints for *E. coli* and *C. koseri *were used.

^*g*^ *E. coli* breakpoints for uncomplicated urinary infections.

^*h*^ *E. coli* breakpoints for uncomplicated urinary infections.

^*i*^ –, no current breakpoints in EUCAST guidelines.

### MLST and SNP analysis

**Fig 2:**
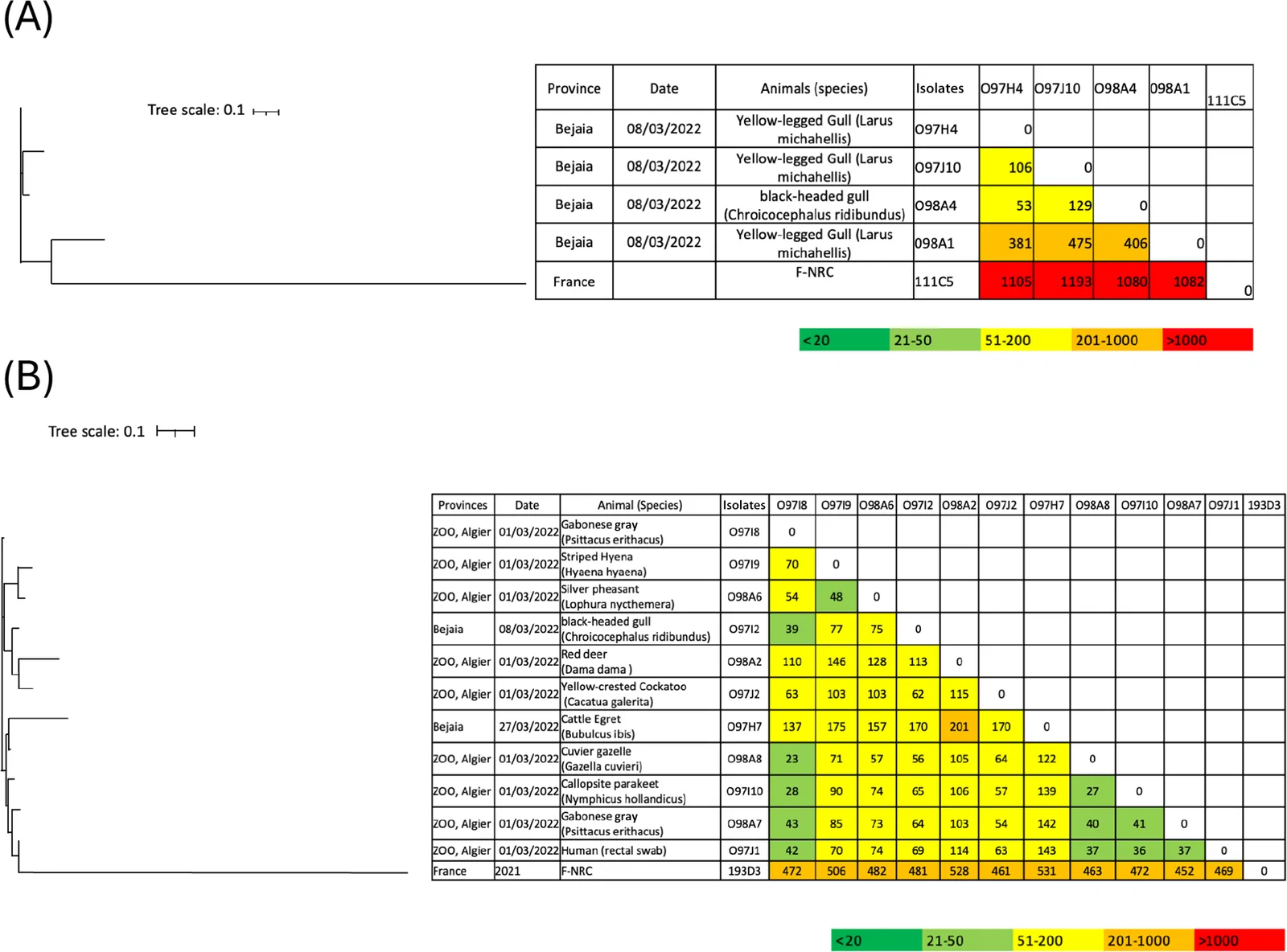
Phylogenetic tree and SNP matrix analysis of (**A**) OXA-48-producing *K. pneumoniae* ST13 and (**B**) *Enterobacter hormaechei hoffmannii* ST145.

Three STs of OXA-48-*Kp* were identified, including ST13 (*n* = 4), ST2674 (*n* = 1), and ST1621 (*n* = 1), while OXA-48-*Kq* belonged to ST2787 and OXA-48-*Km* to ST88 (Table 2). The SNP comparison matrix of the four *Kp* ST13 revealed a minimum of 53 SNP differences (Fig. 2A). These strains were all isolated from wild migratory birds (yellow-legged gull, *n* = 3; and black-headed gull, *n* = 1) from the same region of Bejaia. In addition, *Km* ST88 was also detected in a black-headed gull in Bejaia (Table 2).

OXA-48-*Eh* isolates belonged to two STs: ST286 (*n* = 2) isolated from birds, a Dipper and Wagtail in the city of Jijel (Table 3), and ST145 (*n* = 11) recovered from zoo wild animal droppings and from one zoo animal care worker of the El Hamma Zoo in Algiers. Two closely related *Eh* ST145 (<50 SNPs with those of Algiers) were isolated in Bejaia, a city located 247 km apart from Algiers, from the feces of sedentary breeding (Cattle egret) and migratory (black-headed gull) wild birds, (Table 2), suggesting a possible transmission between these cities. SNP matrix analysis of *Eh* ST145 revealed that some of these isolates were likely related to less than 50 SNPs, especially those isolated in the zoo, including that of one zoo animal care worker (Fig. 2B).

Five different STs were identified among the *Ec* isolates, including ST131 (*n* = 2) expressing OXA-244 from a Capuchin and Elephant of Jijel zoological park, OXA-244-producing *Ec* ST1135 (*n* = 1), OXA-48-producing *Ec* ST38 (*n* = 1) and OXA-181-producing *Ec* ST540 (*n* = 1) from migratory bird droppings of Bejaia, and *Ec* ST227 (*n* = 1) isolated from a Fennec in Jijel’s zoological park. *Cf* ST646 was isolated from a wild bird in Bejaia (Table 2).

### Susceptibility profiles of CPEs

Based on the WGS results, the niches and the sampling sites 18/30 CPEs were further investigated using broth microdilution. All the isolates were resistant to amoxicillin, to amoxicillin/clavulanic acid, piperacillin, piperacillin/tazobactam, ticarcillin, ticarcillin/clavulanic acid, temocillin, and ertapenem, and revealed variable levels of resistance to the other tested antibiotics (Table 3). Eleven of these isolates were multi-susceptible besides β-lactam resistance. All isolates were susceptible to colistin and to newer β-lactam/inhibitor combinations. (Table 3). The three OXA-244 and the OXA-181 *E. coli* isolates were MDR.

### Genetic support of *bla*_OXA-48-like_ carbapenemase genes

Plasmid analysis revealed that 24/30 CPEs contained at least an IncL replicon and a plasmid of c.a. 62 kb likely corresponding to the prototypical IncL pOXA-48 plasmid, while the remaining six *Ec* isolates contained other replicon types and different sized plasmids (Table 2 and Fig. S1) (33, 34). Electroporation experiments revealed transformants with similar resistance profiles (Data not shown) compatible with OXA-48 expression and transfer of a single plasmid of c.a. 62 kb for 24/30 CPEs (Fig. S2) (35). Mapping of the Illumina reads of these 24 CPEs to the prototypical pOXA-48 plasmid revealed nearly 99.99% sequence identity (35).

**Fig 3:**
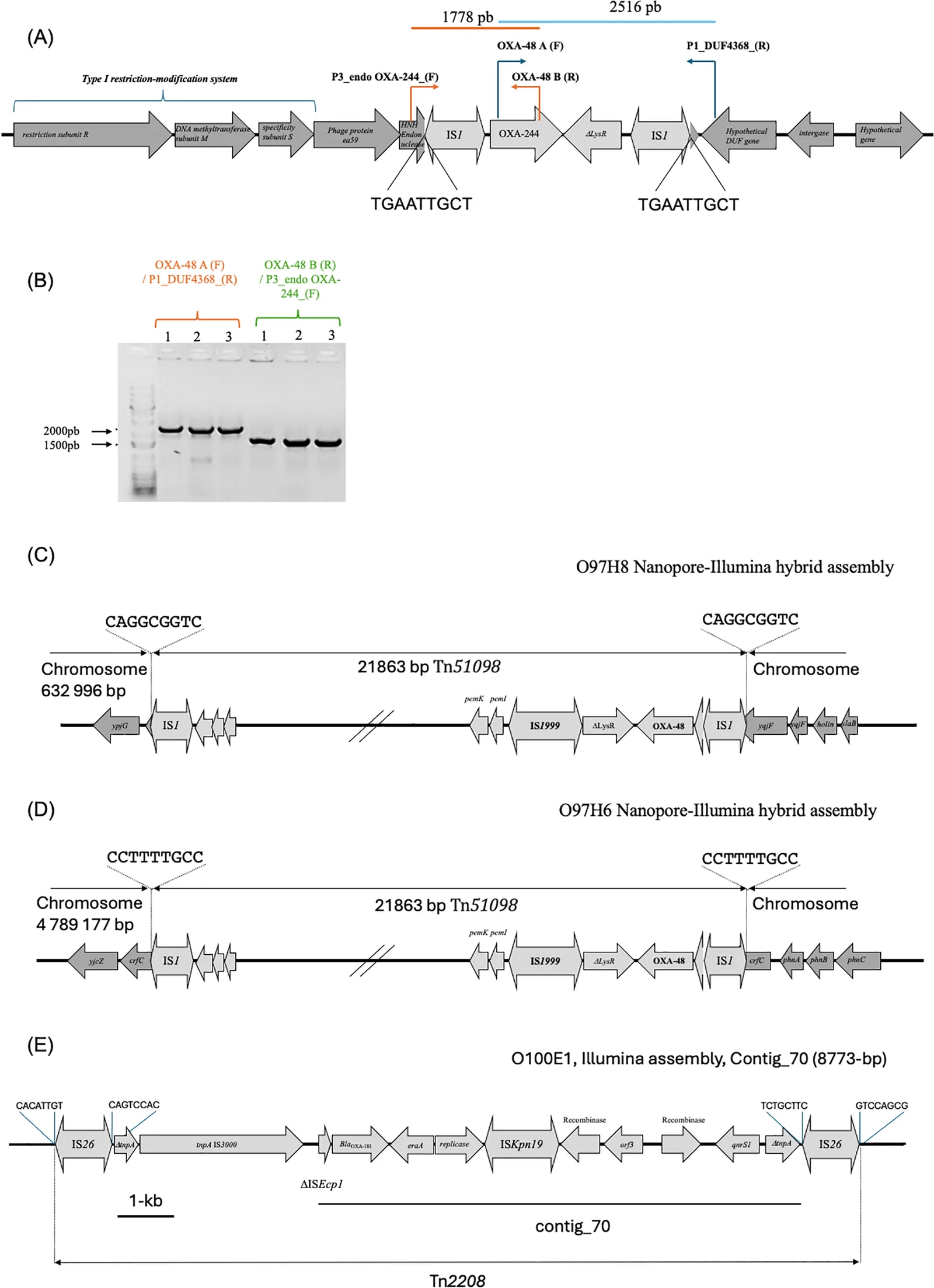
Genetic environment of chromosomally- (**A–D**) and plasmid- (E) encoded *bla*_OXA-48-like_ genes. (A) *bla*_OXA-244_ genes in *E. coli* ST131 O97H9, *E. coli* ST131 O97H10, and *E. coli* ST1135 O97I5; (**B**) Agarose gel revealing PCR products for chromosomal insertion verification; (**C**) *bla*_OXA-48_ gene in *E. coli* ST38 O97H6; (**D**) *bla*_OXA-48_ gene in *E. coli* ST227 O97H8 as revealed by Nanopore-Illumina hybrid assembly; and (**E**) *E. coli* O100E1 ST540 *bla*_OXA-181_ genetic environment. Dark gray boxes represent chromosome-borne genes, while light gray boxes represent either integrated genes from plasmid pOXA-48a (**A–D**) or plasmid-borne genes (**E**). Genes are indicated by arrows and insertion sequences by double arrows. Target site duplications are indicated at the end of insertion sequences.

Despite the presence of multiple replicons, transformation and mating out assays repeatedly failed with the five/six OXA-48-like-producing *Ec*. Mapping of the Illumina reads of the three OXA-244-producing *E. coli* isolates against the genome of an OXA-244-producing *E. coli* (CP050382) (36) revealed a similar genetic organization with the *bla*_OXA-244_ gene embedded in a 3.3 kb in size truncated Tn*51098* inserted in the 3′ end of a chromosomally encoded *HNH endonuclease* gene (Fig. 3A) (7, 8). This genetic organization was confirmed using PCR amplifications of the boundaries of the inserted composite transposon, confirming a chromosomal location in these OXA-244-producers (Fig. 3B).

Nanopore sequencing and hybrid genomic assembly of OXA-48 producing *E. coli* ST38 and ST227 isolates revealed that the *bla*_OXA-48_ gene was located on Tn*51098*, a 21.9 kb composite transposon made of two IS*1* bracketing a DNA fragment derived from the pOXA-48 plasmid (stretching from Δ*tir-pemI* to *korC-orf25*), inserted into two distinct genetic loci (Fig. 3C and D) (8, 33, 37). As recently shown, integration of OXA-48 variants into the chromosome of *E. coli* ST38 depends on a novel antiplasmid system ApsAB that is actively involved in pOXA-48 destabilization (34). ApsAB was present in *E. coli* ST38 but absent from the other 5 *E. coli* genomes. In *Ec* ST540, the *bla*_OXA-181_ gene is carried on an 8.7 kb contig that is 100% identical to the corresponding region of a 51,479 plasmid (CP023897) as revealed by Blast analysis. Mapping Illumina reads against this plasmid used as a reference sequence revealed 100% sequence identity. This IncFII-ColKP3 hybrid plasmid could be electroporated and conjugated into *E. coli*, thus confirming the plasmidic and its self-transferable nature (Fig. 3E; Fig. S1 and S2). Careful analysis of the genetic environment of *bla*_OXA-181_ revealed an upstream-located truncated IS*Ecp1* and a 14,141 bp composite transposon named Tn*2208*, made of two IS*26* elements bracketing ColKP3-derived plasmid sequence carrying *bla*_OXA-181_ gene.

## DISCUSSION

The spread of CPEs is increasingly described worldwide in hospitals, livestock, companion animals, and in environmental settings (37). Their dissemination through wild and captive wildlife remains poorly understood. This study links ecology and antibiotic resistance by analyzing healthy wild zoo animals (38). CPEs were detected in 1.2% (*n* = 18/1,521) of wild animals, 3.0% (*n* = 11/365) of samples from captive animals, and 7.7% (*n* = 1/13) from zoo care workers (Table 1). The presence of CPEs in zoos is rare, while ESBL rates may be high (11%–32%) (13–15, 19–21). In Algeria, livestock animals show a high prevalence of CPEs (26%), whereas it remains low in wild animals (39, 40).

OXA-48-like producing Enterobacterales have predominantly been described in human samples and are considered endemic in hospitals of North African countries such as Algeria (5, 37), with in recent years an increasing description of NDM-producing isolates. In our study, only *bla*_OXA-48-like_ genes were identified, representing 1.53% of all samples examined, demonstrating a widespread dissemination of this resistance mechanism across Algeria (41). OXA-48 producers have already been described in a few cases of companion and wild animals (42); here, CPEs were found in wild animals of three major Algerian provinces: the Bejaia province with 16 CPEs identified from free-ranging wild animals; the province of Algiers with eight CPEs from captive zoo animals and from one healthy animal care worker; and the province of Jijel with five CPEs from free-ranging wild birds (*n* = 2) and zoo animals (*n* = 3; Fig. 1A and Table 1).

The widespread presence of *bla_OXA-48-like_* genes is highlighted by their occurrence in various Enterobacterales species. Among the 30 OXA-48-like producers, 13 were *E. hormaechei* subsp. *hoffmannii* isolates, belonging to ST145 (11 isolates) and ST286 (2 isolates), both reported for the first time in Algeria. *Eh* ST145 isolates were found in various animals (birds and mammals) at El Hamma Zoo in Algiers, including one from an animal care worker, suggesting possible human-to-animal transmission. Our findings indicate the spread of a single OXA-48-producing *Eh* ST145 clone within the zoo, posing a potential health risk to workers and visitors (43). *Eh* ST145 isolates have also been found in free-ranging birds (Cattle Egret and black-headed gull) in Bejaia, a city located 247 km from Algiers (Fig. 1A). These isolates had 23–201 SNP differences with those of Algiers but presented more than 450 SNPs with a human isolate from the French National Reference Center for CPEs used as control. These results suggest an epidemiological link between these isolates and a spread beyond the zoo of Algiers. Transmission of these strains may have occurred through sharing a common diet, but other pathways, such as housing near each other or sharing the same animal care worker, may have contributed to the clonal expansion. Wild birds such as the cattle egret and black-headed gull may play a role in the spread of *Eh* ST145 across the country and even beyond as suggested for ESBL producers (44). OXA-48-producing isolates of *Eh ST145* are rare. While VIM-producing *E. hormaechei subsp. hoffmannii* have been described from human origin, *Eh* ST145 corresponded to only 4.7% of the total isolates, and none to ST286 (45). In a recent study, WGS of OXA-48-producing *E. hormaechei* that were repeatedly identified in infections in companion animals hospitalized at a Swiss veterinary clinic corresponded to *E. hormaechei subsp. xiangfangensis* ST114 (*n* = 6) and ST418 (*n* = 2), and *E. hormaechei subsp. hoffmannii* ST78 (*n* = 1) (46).

*Klebsiella* species are the second most reported OXA-48-producing Enterobacterales in our study, with *Kp* (6 isolates), *Kq* (2 isolates), and *Km* (1 isolate). *Kp* ST13 was the predominant ST (4/9) that was recovered from migratory yellow-legged gull and black-headed gull in the province of Bejaia (Fig. 1). A recent study on the genomic population structure of human *Kp* isolates from Portugal revealed that *Kp* ST13-producing KPC-3 is among the four disseminating high-risk clones in that country, and these isolates have spread to France and the Netherlands (47, 48). ST13 has previously been described in the Bejaia geographical area in various ecological niches, including wild boar, fish, cattle, foodstuffs, but also from fecal samples of mothers and newborns hospitalized in two maternities in Bejaia and Tizi Ouzou, but not yet in yellow-legged or black-headed gulls (5, 13). These observations highlight the widespread presence of *Kp* ST13 in environmental reservoirs in various geographic areas, emphasizing its ecological adaptability and importance in the dissemination of carbapenemase resistance genes. In a recent systematic review that explored the prevalence of *Klebsiella* spp. bacteria in wild animals on a global level, none of the STs identified in this study have been described elsewhere (49).

OXA-48-producing *Kp* ST2674, found in a magot monkey in Bejaia (Fig. 1A), has previously been reported as the cause of severe septicemia in patients in intensive care units in Russia (50). OXA-48-producing *Kq* ST2787 that was isolated from the droppings of a yellow-legged gull in Bejaia has never been described so far (Fig. 1A); only one KPC-2-producing *Kq* ST2787 isolate has previously been described in 2018 from raw sewage of a wastewater treatment plant in Argentina (51). Similarly, OXA-48-producing *Km* ST88 and *Kp* ST1621, which were isolated from the droppings of the house swallow and the black-headed gull, respectively, in Bejaia, have never been described so far.

Even though five distinct STs of OXA-48-like-producing *Ec* isolates were identified, five correspond to human high-risk clones (ST38, ST227, ST540, and 2 ST131) that were MDR. Indeed, *E. coli* ST131 is responsible for the spread of *bla*_CTX-M-15_ ESBL gene worldwide (52). Here, both *E. coli* ST131 isolates that were recovered from a Capuchin and an elephant of the zoological park of Jijel co-expressed *bla*_OXA-244_ and *bla*_CTX-M-15_ genes. OXA-48-producing *Ec* ST131 was initially detected in patients hospitalized at a military hospital in Algiers, and OXA-244-producing *Ec* ST131 was reported in three patients across two tertiary hospitals in southwest Germany, as well as in surface waters within an urban area in northern Italy (3, 53, 54). A recent work done in 17 countries of the European Economic Area revealed the acquisition by *Ec* ST131 of *bla*_OXA-244_ (*n* = 230/594) and *bla*_OXA-48_ (*n* = 224/594), with a rapid increase in isolates carrying *bla*_OXA-244_ since 2021. The increasing detection of carbapenemase genes in the *Ec* high-risk lineage ST131 is a public health concern, as these bacteria are responsible for severe infections in humans and carbapenems, the treatment of choice (55). The epidemiological impact of their detection in zoo animals is a matter of great concern. Another OXA-244-producing *Ec* ST1135 that co-produced CMY-2 and CTX-M-15 was isolated from a black-headed gull in the province of Bejaia. NDM-5-producing *Ec* ST1135 was recently detected for the first time from a neonatal intensive care unit in Kolkata, India (56). OXA-48-producing *Ec* ST38 (*n* = 1), isolated from a Cattle Egret in our study, has previously been detected in various bird species but also in human samples in Algeria, suggesting an endemic multi-sectorial spread (57, 58). A recent study that compared wild bird-originated *Ec* ST38 genome sequences with publicly available genomic data derived from humans and environments revealed frequent exchanges between these compartments (59). It further suggested that carbapenem-resistant *Ec* ST38 clones in wild birds were likely acquired from the local environment, but intercontinental dispersal of OXA-48-producing *Ec* ST38 clones through gulls between Alaska and Turkey has also been suggested (59). In another study, the *bla*_OXA-48_ gene was found in 24.32% (9/37) of carbapenem-resistant *Ec* isolates recovered from 137 fecal samples of seagulls from Italy (60). Interestingly, in this country, mostly KPC and NDM are isolated in human samples, suggesting that OXA-48-*Ec* isolates may be present in the environment the seagulls are living, or that they have acquired them abroad (60, 61).

The OXA-48-producing *Ec* ST227 isolate, detected in the feces of a Fennec, is commonly reported worldwide, from human and environmental sources. Finally, *Ec* ST540 identified in a gull from Bejaia has already been reported in ESBL‐producing *E. coli* from gulls in Chile, Sweden, and Australia (44, 62, 63), from captive Black Bears from China (64), from cattle in Italy (65), from cattle, sheep, and camel meat in Tunisia (66), and from humans in Jordan (67), and one OXA-48-producing *Ec* was characterized from a hospitalized patient in Spain in 2013 (68). However, OXA-181-producing *Ec* ST540 has never been described neither in animals nor in humans so far. Similarly, OXA-48-producing *Cf* ST527 isolated from our black-headed gull has never been reported from animals worldwide so far.

To further understand the spread of OXA-48-like-producing Enterobacterales among wildlife, we investigated the *bla*_OXA-48-like_ genetic support. In 24/30 isolates, the *bla*_OXA-48_ gene was carried by the IncL-type plasmid pOXA-48a, which is globally reported to be associated with *bla*_OXA-48_ genes and with high transfer capacity among different Enterobacterales species (Fig. S1 and S2) (37). Interestingly, in the five/six *Ec* isolates, the *bla*_OXA-48-like_ gene was chromosome borne. The two OXA-48-producing isolates contained an integrated composite transposon, Tn*51098* (7, 8), inserted at two different loci of the *Ec* chromosome. The *bla*_OXA-244_ gene was initially also reported to be embedded in Tn*51098* integrated into the chromosome of *Ec* ST38, but recent work revealed that in some clinical isolates from France, the *bla*_OXA-244_ gene was embedded in a 3.3 kb in size truncated Tn*51098* inserted in the 3′ end of an HNH endonuclease gene (Fig. 3A and B) (7, 8). The three OXA-244-producing *Ec* isolates had the same genetic structure integrated at the same loci. The effect on the clonal dissemination of this reduced Tn*51098* (e.g., better fitness) and its integration at the same loci remains undetermined. While it has been shown that integration of OXA-48 variants into the chromosome of *Ec* ST38 depends on the ApsAB antiplasmid system (34), for the other four *E. coli* isolates, the driver for plasmid integration is not determined. In *Ec* O100E1 ST540, isolated from a black-headed gull, the *bla*_OXA-181_ gene has been mobilized through an IS26-borne composite transposon, Tn2208, that is carried by a 51.4 kb self-transferable plasmid (Fig. 3E; 1S and 2S). Unlike the *bla*_OXA-48_ gene, which is frequently associated with the Tn*1999* transposon, the *bla*_OXA-181_ gene is frequently associated with IS*Ecp1* (37, 69, 70), but here, only a truncated form of IS*Ecp1* is found, suggesting further diversification of genetic structures along with their dispersal.

### Conclusion

This study suggests a high prevalence and widespread dissemination of OXA-48-like-producing Enterobacterales in various ecological niches beyond clinical settings in Algeria. This dissemination is mainly due to the success of the IncL pOXA-48 plasmid among various Enterobacterales species, to the clonal dissemination of *E. hormaechei hoffmannii* (ST145) and *K. pneumoniae* (ST13) isolates, and to the cross-sectorial spread of high-risk *E. coli* clones.

At present, it remains unclear whether these CPEs result from spillover from humans or whether wildlife may act as intermediate hosts between environmental bacteria such as *Shewanella*, which naturally carry *bla*_OXA-48_ genes, and human isolates. While high-risk *E. coli* clones likely originate from humans, the situation is less clear for *Enterobacter hormaechei* subsp. *hoffmannii*, which is commonly found in animals. Nevertheless, free-ranging and captive wildlife play now a key role in promoting transmission between animals and to humans and thus may be considered a reservoir of CPEs. This is particularly true for wild animals that adapt to diverse environments and interact directly or indirectly with both humans and livestock (71). Seagulls, for example, accounted for 12 of 30 CPE cases, highlighting their potential role as dissemination vectors, along with migratory wild birds that may facilitate the international spread of antibiotic resistance (71).

The trafficking of wild animals from regions with high MDR prevalence to less affected areas further exacerbates this phenomenon (72). The impact of CPEs in wildlife on human health remains poorly understood, although they may cause infections, particularly in low and middle income countries (73). Our findings highlight the urgent need for surveillance and research on CPE transmission in ecosystems closely linked to human activity, as well as interventions to mitigate the dissemination of antimicrobial resistance throughout the environment, best exemplified by the acquisition of carbapenem resistance by birds.

## SUPPLEMENTAL MATERIAL

Supplemental tables and figures spectrum.00432-26-s0001.docx Tables S1–S3; Fig. S1 and S2.

ASM does not own the copyrights to Supplemental Material that may be linked to, or accessed through, an article. The authors have granted ASM a non-exclusive, world-wide license to publish the Supplemental Material files. Please contact the corresponding author directly for reuse.

## References

[1] Nordmann P, Cuzon G, Naas T. 2009. The real threat of *Klebsiella pneumoniae* carbapenemase-producing bacteria. Lancet Infect Dis 9:228–236. doi:10.1016/S1473-3099(09)70054-4 19324295

[2] Nordmann P, Naas T, Poirel L. 2011. Global spread of carbapenemase-producing *Enterobacteriaceae*. Emerg Infect Dis 17:1791–1798. doi:10.3201/eid1710.110655 22000347 PMC3310682

[3] Agabou A, Pantel A, Ouchenane Z, Lezzar N, Khemissi S, Satta D, Sotto A, Lavigne JP. 2014. First description of OXA-48-producing *Escherichia coli* and the pandemic clone ST131 from patients hospitalised at a military hospital in Algeria. Eur J Clin Microbiol Infect Dis 33:1641–1646. doi:10.1007/s10096-014-2122-y 24792128

[4] Bourafa N, Chaalal W, Bakour S, Lalaoui R, Boutefnouchet N, Diene SM, Rolain JM. 2018. Molecular characterization of carbapenem-resistant gram-negative bacilli clinical isolates in algeria. Infect Drug Resist 11:735–742. doi:10.2147/IDR.S150005 29844691 PMC5961646

[5] Mairi A, Pantel A, Sotto A, Lavigne JP, Touati A. 2018. OXA-48-like carbapenemases producing *Enterobacteriaceae* in different niches. Eur J Clin Microbiol Infect Dis 37:587–604. doi:10.1007/s10096-017-3112-7 28990132

[6] Emeraud C, Biez L, Girlich D, Jousset AB, Naas T, Bonnin RA, Dortet L. 2020. Screening of OXA-244 producers, a difficult-to-detect and emerging OXA-48 variant? J Antimicrob Chemother 75:2120–2123. doi:10.1093/jac/dkaa155 32363407

[7] Emeraud C, Girlich D, Bonnin RA, Jousset AB, Naas T, Dortet L. 2021. Emergence and polyclonal dissemination of OXA-244-producing *Escherichia coli*, France. Emerg Infect Dis 27:1206–1210. doi:10.3201/eid2704.204459 33755001 PMC8007313

[8] Hoyos-Mallecot Y, Naas T, Bonnin RA, Patino R, Glaser P, Fortineau N, Dortet L. 2017. OXA-244-producing *Escherichia coli* isolates, a challenge for clinical microbiology laboratories. Antimicrob Agents Chemother 61. doi:10.1128/AAC.00818-17 PMC5571363 28674064

[9] Naas T, Oueslati S, Bonnin RA, Dabos ML, Zavala A, Dortet L, Retailleau P, Iorga BI. 2017. Beta-lactamase database (BLDB) - structure and function. J Enzyme Inhib Med Chem 32:917–919. doi:10.1080/14756366.2017.1344235 28719998 PMC6445328

[10] Rima M, Emeraud C, Bonnin RA, Gonzalez C, Dortet L, Iorga BI, Oueslati S, Naas T. 2021. Biochemical characterization of OXA-244, an emerging OXA-48 variant with reduced β-lactam hydrolytic activity. J Antimicrob Chemother 76:2024–2028. doi:10.1093/jac/dkab142 33993262

[11] McEwen SA, Collignon PJ. 2018. Antimicrobial resistance: a one health perspective. Microbiol Spectr 6. doi:10.1128/microbiolspec.arba-0009-2017 PMC11633550 29600770

[12] Plaza-Rodriguez C, Alt K, Grobbel M, Hammerl JA, Irrgang A, Szabo I, Stingl K, Schuh E, Wiehle L, Pfefferkorn B, Naumann S, Kaesbohrer A, Tenhagen BA. 2020. Wildlife as sentinels of antimicrobial resistance in Germany? Front Vet Sci:627821. doi:10.3389/fvets.2020.627821 33585611 PMC7873465

[13] Bachiri T, Bakour S, Lalaoui R, Belkebla N, Allouache M, Rolain JM, Touati A. 2018. Occurrence of carbapenemase-producing *Enterobacteriaceae* isolates in the wildlife: first report of OXA-48 in wild boars in Algeria. Microb Drug Resist 24:337–345. doi:10.1089/mdr.2016.0323 28799835

[14] Gharout-Sait A, Touati A, Ahmim M, Brasme L, Guillard T, Agsous A, Champs C. 2019. Occurrence of carbapenemase-producing *Klebsiella pneumoniae* in bat guano. Microb Drug Resist 25:1057–1062. doi:10.1089/mdr.2018.0471 31021173

[15] Loucif L, Chelaghma W, Bendjama E, Bouaziz A, Cherak Z, Serrar W, Mohammed FS, Rolain JM. 2022. Urban pigeons as a reservoir of carbapenem resistant Enterobacterales: first report of OXA-48-producing *Klebsiella pneumoniae*. New Microbes New Infect 47:100981. doi:10.1016/j.nmni.2022.100981 35712261 PMC9194841

[16] Conrad CC, Stanford K, Narvaez-Bravo C, Neumann NF, Munns K, Tymensen L, Jokinen C, McAllister TA. 2018. Zoonotic fecal pathogens and antimicrobial resistance in Canadian petting zoos. Microorganisms 6:70. doi:10.3390/microorganisms6030070 30012975 PMC6164440

[17] Erdozain G, KuKanich K, Chapman B, Powell D. 2013. Observation of public health risk behaviours, risk communication and hand hygiene at kansas and missouri petting zoos--2010-2011. Zoonoses Public Health 60:304–310. doi:10.1111/j.1863-2378.2012.01531.x 22846186

[18] Goode B, O’Reilly C, Dunn J, Fullerton K, Smith S, Ghneim G, Keen J, Durso L, Davies M, Montgomery S. 2009. Outbreak of *Escherichia coli* O157:H7 infections after petting zoo visits, North Carolina State Fair, October-November 2004. Arch Pediatr Adolesc Med 163:42–48. doi:10.1001/archpediatrics.2008.525 19124702

[19] Dobiasova H, Dolejska M, Jamborova I, Brhelova E, Blazkova L, Papousek I, Kozlova M, Klimes J, Cizek A, Literak I. 2013. Extended-spectrum beta-lactamase and fluoroquinolone resistance genes and plasmids among *Escherichia coli* isolates from zoo animals, Czech Republic. FEMS Microbiol Ecol 85:604–611. doi:10.1111/1574-6941.12149 23679004

[20] Gottling J, Heckel JO, Hotzel H, Fruth A, Pfeifer Y, Henning K, Kopp P, Mertens-Scholz K, Rietschel W, Pfeffer M. 2022. Zoonotic bacteria in clinically healthy goats in petting zoo settings of zoological gardens in Germany. Zoonoses Public Health 69:333–343. doi:10.1111/zph.12922 35229466

[21] Shnaiderman-Torban A, Steinman A, Meidan G, Paitan Y, Abu Ahmad W, Navon-Venezia S. 2019. Petting zoo animals as an emerging reservoir of extended-spectrum β-lactamase and AmpC-producing *Enterobacteriaceae*. Front Microbiol 10:2488. doi:10.3389/fmicb.2019.02488 31736921 PMC6831544

[22] Wang Y, He T, Han J, Wang J, Foley SL, Yang G, Wan S, Shen J, Wu C. 2012. Prevalence of ESBLs and PMQR genes in fecal *Escherichia coli* isolated from the non-human primates in six zoos in China. Vet Microbiol 159:53–59. doi:10.1016/j.vetmic.2012.03.009 22487457

[23] Daaboul D, Oueslati S, Rima M, Kassem II, Mallat H, Birer A, Girlich D, Hamze M, Dabboussi F, Osman M, Naas T. 2023. The emergence of carbapenemase-producing *Enterobacterales* in hospitals: a major challenge for a debilitated healthcare system in Lebanon. Front Public Health 11:1290912. doi:10.3389/fpubh.2023.1290912 38074718 PMC10699444

[24] Boutal H, Vogel A, Bernabeu S, Devilliers K, Creton E, Cotellon G, Plaisance M, Oueslati S, Dortet L, Jousset A, Simon S, Naas T, Volland H. 2018. A multiplex lateral flow immunoassay for the rapid identification of NDM-, KPC-, IMP- and VIM-type and OXA-48-like carbapenemase-producing *Enterobacteriaceae*. J Antimicrob Chemother 73:909–915. doi:10.1093/jac/dkx521 29365094 PMC5890661

[25] Dortet L, Agathine A, Naas T, Cuzon G, Poirel L, Nordmann P. 2015. Evaluation of the RAPIDEC CARBA NP, the rapid CARB Screen and the carba NP test for biochemical detection of carbapenemase-producing *Enterobacteriaceae*. J Antimicrob Chemother 70:3014–3022. doi:10.1093/jac/dkv213 26260131

[26] Poirel L, Heritier C, Tolun V, Nordmann P. 2004. Emergence of oxacillinase-mediated resistance to imipenem in *Klebsiella pneumoniae**.* Antimicrob Agents Chemother 48:15–22. doi:10.1128/AAC.48.1.15-22.2004 14693513 PMC310167

[27] Potron A, Poirel L, Dortet L, Nordmann P. 2016. Characterisation of OXA-244, a chromosomally-encoded OXA-48-like β-lactamase from *Escherichia coli*. Int J Antimicrob Agents 47:102–103. doi:10.1016/j.ijantimicag.2015.10.015 26655033

[28] Coll F, Raven KE, Knight GM, Blane B, Harrison EM, Leek D, Enoch DA, Brown NM, Parkhill J, Peacock SJ. 2020. Definition of a genetic relatedness cutoff to exclude recent transmission of meticillin-resistant *Staphylococcus aureus*: a genomic epidemiology analysis. Lancet Microbe 1:e328–e335. doi:10.1016/S2666-5247(20)30149-X 33313577 PMC7721685

[29] Cuzon G, Naas T, Nordmann P. 2011. Functional characterization of Tn4401, a Tn3-based transposon involved in *blaKPC* gene mobilization. Antimicrob Agents Chemother 55:5370–5373. doi:10.1128/AAC.05202-11 21844325 PMC3195030

[30] Kieser T. 1984. Factors affecting the isolation of CCC DNA from *Streptomyces lividans* and *Escherichia coli**.* Plasmid 12:19–36. doi:10.1016/0147-619x(84)90063-5 6387733

[31] Dabos L, Raczynska JE, Bogaerts P, Zavala A, Girlich D, Bonnin RA, Dortet L, Peyrat A, Retailleau P, Iorga BI, Jaskolski M, Glupczynski Y, Naas T. 2023. Structural and biochemical features of OXA-517: a carbapenem and expanded-spectrum cephalosporin hydrolyzing OXA-48 variant. Antimicrob Agents Chemother 67:e0109522. doi:10.1128/aac.01095-22 36648230 PMC9933634

[32] Philippon LN, Naas T, Bouthors AT, Barakett V, Nordmann P. 1997. OXA-18, a class D clavulanic acid-inhibited extended-spectrum beta-lactamase from *Pseudomonas aeruginosa*. Antimicrob Agents Chemother 41:2188–2195. doi:10.1128/AAC.41.10.2188 9333046 PMC164091

[33] Patino-Navarrete R, Rosinski-Chupin I, Cabanel N, Zongo PD, Hery M, Oueslati S, Girlich D, Dortet L, Bonnin RA, Naas T, Glaser P. 2022. Specificities and commonalities of carbapenemase-producing *Escherichia coli* isolated in France from 2012 to 2015. mSystems:e0116921. doi:10.1128/msystems.01169-21 35014866 PMC8751382

[34] Zongo PD, Cabanel N, Royer G, Depardieu F, Hartmann A, Naas T, Glaser P, Rosinski-Chupin I. 2024. An antiplasmid system drives antibiotic resistance gene integration in carbapenemase-producing *Escherichia coli* lineages. Nat Commun 15:4093. doi:10.1038/s41467-024-48219-y 38750030 PMC11096173

[35] Poirel L, Bonnin RA, Nordmann P. 2012. Genetic features of the widespread plasmid coding for the carbapenemase OXA-48. Antimicrob Agents Chemother 56:559–562. doi:10.1128/AAC.05289-11 22083465 PMC3256075

[36] Chudejova K, Kraftova L, Mattioni Marchetti V, Hrabak J, Papagiannitsis CC, Bitar I. 2021. Genetic plurality of OXA/NDM-encoding features characterized from *Enterobacterales* recovered from Czech hospitals. Front Microbiol 12:641415. doi:10.3389/fmicb.2021.641415 33633720 PMC7900173

[37] Pitout JDD, Peirano G, Kock MM, Strydom KA, Matsumura Y. 2019. The global ascendency of OXA-48-type carbapenemases. Clin Microbiol Rev 33:e00102-19. doi:10.1128/CMR.00102-19 31722889 PMC6860007

[38] De Witte C, Vereecke N, Theuns S, De Ruyck C, Vercammen F, Bouts T, Boyen F, Nauwynck H, Haesebrouck F. 2021. Presence of broad-spectrum beta-lactamase-producing *Enterobacteriaceae* in zoo mammals. Microorganisms 9. doi:10.3390/microorganisms9040834 PMC8070755 33919869

[39] Guerra B, Fischer J, Helmuth R. 2014. An emerging public health problem: acquired carbapenemase-producing microorganisms are present in food-producing animals, their environment, companion animals and wild birds. Vet Microbiol 171:290–297. doi:10.1016/j.vetmic.2014.02.001 24629777

[40] Kock R, Daniels-Haardt I, Becker K, Mellmann A, Friedrich AW, Mevius D, Schwarz S, Jurke A. 2018. Carbapenem-resistant *Enterobacteriaceae* in wildlife, food-producing, and companion animals: a systematic review. Clin Microbiol Infect 24:1241–1250. doi:10.1016/j.cmi.2018.04.004 29654871

[41] Bougouizi A, Chekroud Z, Rahab H, Boumegoura A, Touati A. 2024. Prevalence and characterization of carbapenem-resistant enterobacterales among inpatients and outpatients in Skikda, Algeria. J Infect Dev Ctries 18:383–390. doi:10.3855/jidc.18263 38635605

[42] Aslam B, Khurshid M, Arshad MI, Muzammil S, Rasool M, Yasmeen N, Shah T, Chaudhry TH, Rasool MH, Shahid A, Xueshan X, Baloch Z. 2021. Antibiotic resistance: one health one world outlook. Front Cell Infect Microbiol 11:771510. doi:10.3389/fcimb.2021.771510 34900756 PMC8656695

[43] Smet A, Martel A, Persoons D, Dewulf J, Heyndrickx M, Herman L, Haesebrouck F, Butaye P. 2010. Broad-spectrum β-lactamases among *Enterobacteriaceae* of animal origin: molecular aspects, mobility and impact on public health. FEMS Microbiol Rev 34:295–316. doi:10.1111/j.1574-6976.2009.00198.x 20030731

[44] Mukerji S, Gunasekera S, Dunlop JN, Stegger M, Jordan D, Laird T, Abraham RJ, Barton M, O’Dea M, Abraham S. 2020. Implications of foraging and interspecies interactions of birds for carriage of *Escherichia coli* strains resistant to critically important antimicrobials. Appl Environ Microbiol 86:e01610-20. doi:10.1128/AEM.01610-20 32801178 PMC7531969

[45] Emeraud C, Petit C, Gauthier L, Bonnin RA, Naas T, Dortet L. 2022. Emergence of VIM-producing *Enterobacter cloacae* complex in France between 2015 and 2018. J Antimicrob Chemother 77:944–951. doi:10.1093/jac/dkab471 35045171

[46] Dona V, Nordmann P, Kittl S, Schuller S, Bouvier M, Poirel L, Endimiani A, Perreten V. 2023. Emergence of OXA-48-producing *Enterobacter hormaechei* in a Swiss companion animal clinic and their genetic relationship to clinical human isolates. J Antimicrob Chemother 78:2950–2960. doi:10.1093/jac/dkad337 37923369

[47] Elias R, Spadar A, Hendrickx APA, Bonnin RA, Dortet L, Pinto M, Phelan JE, Portugal I, Campino S, da Silva GJ, Clark TG, Duarte A, Perdigao J. 2023. Emergence of KPC-3- and OXA-181-producing ST13 and ST17 *Klebsiella pneumoniae* in Portugal: genomic insights on national and international dissemination. J Antimicrob Chemother 78:1300–1308. doi:10.1093/jac/dkad093 36999363

[48] Mendes G, Ramalho JF, Bruschy-Fonseca A, Lito L, Duarte A, Melo-Cristino J, Caneiras C. 2022. Whole-genome sequencing enables molecular characterization of non-clonal group 258 high-risk clones (ST13, ST17, ST147 and ST307) among carbapenem-resistant *Klebsiella pneumoniae* from a tertiary university hospital centre in Portugal. Microorganisms 10:416. doi:10.3390/microorganisms10020416 35208876 PMC8875758

[49] Quintelas M, Silva V, Araújo S, Tejedor-Junco MT, Pereira JE, Igrejas G, Poeta P. 2024. *Klebsiella* in wildlife: clonal dynamics and antibiotic resistance profiles, a systematic review. Pathogens 13. doi:10.3390/pathogens13110945 PMC11597104 39599498

[50] Fursova NK, Astashkin EI, Ershova ON, Aleksandrova IA, Savin IA, Novikova TS, Fedyukina GN, Kislichkina AA, Fursov MV, Kuzina ES, Biketov SF, Dyatlov IA. 2021. Multidrug-resistant *Klebsiella pneumoniae* causing severe infections in the neuro-ICU. Antibiotics (Basel). doi:10.3390/antibiotics10080979 PMC8389041 34439029

[51] Ghiglione B, Haim MS, Penzotti P, Brunetti F, G DAG, Di Conza J, Figueroa-Espinosa R, Nunez L, Razzolini MTP, Fuga B, Esposito F, Vander Horden M, Lincopan N, Gutkind G, Power P, Dropa M. 2021. Characterization of emerging pathogens carrying blaKPC-2. Front Cell Infect Microbiol:722536. doi:10.3389/fcimb.2021.722536 34504809 PMC8421773

[52] Doi Y, Iovleva A, Bonomo RA. 2017. The ecology of extended-spectrum β-lactamases (ESBLs) in the developed world. J Travel Med 24:S44–S51. doi:10.1093/jtm/taw102 28521000 PMC5731446

[53] AbuAlshaar A, Piazza A, Mercato A, Marchesini F, Mattioni Marchetti V, Bitar I, Hrabak J, Spalla M, Pilla G, Sconfietti R, Migliavacca R. 2022. Oxa-244-producing ST131 *Escherichia coli* from surface and groundwaters of pavia urban area (Po Plain, northern Italy). Front Microbiol 13:920319. doi:10.3389/fmicb.2022.920319 35756019 PMC9225575

[54] Welker S, Boutin S, Miethke T, Heeg K, Nurjadi D. 2020. Emergence of carbapenem-resistant ST131 *Escherichia coli* carrying blaOXA-244 in Germany, 2019 to 2020. Euro Surveill 25. doi:10.2807/1560-7917.ES.2020.25.46.2001815 PMC7678038 33213685

[55] Kohlenberg A, Svartstrom O, Apfalter P, Hartl R, Bogaerts P, Huang TD, Chudejova K, Malisova L, Eisfeld J, Sandfort M, et al.. 2024. Emergence of *Escherichia coli* ST131 carrying carbapenemase genes. European Union/European Economic Area. doi:10.2807/1560-7917.ES.2024.29.47.2400727 PMC11583312 39574387

[56] Rao A, Naha S, Bhattacharjee A, Chattopadhyay P, Dutta S, Basu S. 2023. Plasmid-mediated AmpC in *Klebsiella pneumoniae* and *Escherichia col*i from septicaemic neonates: diversity, transmission and phenotypic detection. J Glob Antimicrob Resist 34:9–14. doi:10.1016/j.jgar.2023.05.012 37328061

[57] Mairi A, Pantel A, Ousalem F, Sotto A, Touati A, Lavigne JP. 2019. Oxa-48-producing *Enterobacterales* in different ecological niches in Algeria: clonal expansion, plasmid characteristics and virulence traits. J Antimicrob Chemother 74:1848–1855. doi:10.1093/jac/dkz146 30993328

[58] Mairi A, Touati A, Ait Bessai S, Boutabtoub Y, Khelifi F, Sotto A, Lavigne JP, Pantel A. 2019. Carbapenemase-producing *Enterobacteriaceae* among pregnant women and newborns in Algeria: prevalence, molecular characterization, maternal-neonatal transmission, and risk factors for carriage. Am J Infect Control 47:105–108. doi:10.1016/j.ajic.2018.07.009 30220617

[59] Ahlstrom CA, Woksepp H, Sandegren L, Ramey AM, Bonnedahl J. 2023. Exchange of carbapenem-resistant *Escherichia coli* sequence type 38 intercontinentally and among wild bird, human, and environmental niches. Appl Environ Microbiol 89:e0031923. doi:10.1128/aem.00319-23 37195171 PMC10304903

[60] Cagnoli G, Bertelloni F, Ceccherelli R, Ebani VV. 2024. Antimicrobial resistance and pathotypes of *Escherichia coli* isolates from yellow-legged seagulls (*Larus michahellis)* in central Italy. Animals (Basel) 14. doi:10.3390/ani14213048 PMC11545632 39518773

[61] De Vita E, De Angelis L, Arzilli G, Baglivo F, Barnini S, Vecchione A, Baggiani A, Rizzo C, Porretta AD Teams A 2023. Investigating resistance to carbapenems in *Enterobacterales*: a descriptive epidemiological study of 2021 screening in an Italian teaching hospital. Pathogens 12:1140. doi:10.3390/pathogens12091140 PMC10535761 37764948

[62] Atterby C, Börjesson S, Ny S, Järhult JD, Byfors S, Bonnedahl J. 2017. ESBL-producing *Escherichia coli* in Swedish gulls-a case of environmental pollution from humans? PLoS One 12:e0190380. doi:10.1371/journal.pone.0190380 29284053 PMC5746268

[63] Hernandez J, Johansson A, Stedt J, Bengtsson S, Porczak A, Granholm S, Gonzalez-Acuna D, Olsen B, Bonnedahl J, Drobni M. 2013. Characterization and comparison of extended-spectrum β-lactamase (ESBL) resistance genotypes and population structure of *Escherichia coli* isolated from Franklin’s gulls (*Leucophaeus pipixcan*) and humans in Chile. PLoS One 8:e76150. doi:10.1371/journal.pone.0076150 24098774 PMC3786981

[64] Lei X, Che M, Zhou Y, Pan S, Yang X, Liu S, Laghari I, Wu M, Han R, Li X, Zhou L, Peng G, Liu H, Zhou Z, Zhang K, Zhong Z. 2025. ESBL-producing *E. coli* in captive black bears: molecular characteristics and risk of dissemination. Vet Sci. doi:10.3390/vetsci12111085 PMC12656830 41295723

[65] Di Marcantonio L, Chiatamone Ranieri S, Toro M, Marchegiano A, Cito F, Sulli N, Del Matto I, Di Lollo V, Alessiani A, Foschi G, Platone I, Paoletti M, D’Alterio N, Garofolo G, Janowicz A. 2025. Comprehensive regional study of ESBL *Escherichia coli*: genomic insights into antimicrobial resistance and inter-source dissemination of ESBL genes. Front Microbiol 16:1595652. doi:10.3389/fmicb.2025.1595652 40556893 PMC12185426

[66] Hmidi I, Souguir M, Metayer V, Drapeau A, Francois P, Madec JY, Haenni M, Mansour W. 2025. Characterization of *Enterobacterales* resistant to extended-spectrum cephalosporins isolated from meat in Tunisia. J Food Prot 88:100610. doi:10.1016/j.jfp.2025.100610 40885399

[67] Zueter AM, Mharib T, Shqair D, Al-Tamimi M, Sawan HM, Zaiter A, Albalawi H, Al Balawi D. 2024. Multi-locus sequence typing of *Escherichia coli* isolated from clinical samples in Jordan. J Infect Dev Ctries 18:571–578. doi:10.3855/jidc.18810 38728632

[68] Gijon D, Tedim AP, Valverde A, Rodríguez I, Morosini MI, Coque TM, Manrique M, Pareja E, Tobes R, Ruiz-Garbajosa P, Cantón R. 2020. Early OXA-48-producing enterobacterales isolates recovered in a Spanish hospital reveal a complex introduction dominated by sequence type 11 (ST11) and ST405 *Klebsiella pneumoniae* clones. mSphere 5. doi:10.1128/mSphere.00080-20 PMC7142293 32269151

[69] Aubert D, Naas T, Heritier C, Poirel L, Nordmann P. 2006. Functional characterization of IS1999, an IS4 family element involved in mobilization and expression of beta-lactam resistance genes. J Bacteriol 188:6506–6514. doi:10.1128/JB.00375-06 16952941 PMC1595497

[70] Potron A, Nordmann P, Lafeuille E, Al Maskari Z, Al Rashdi F, Poirel L. 2011. Characterization of OXA-181, a carbapenem-hydrolyzing class D beta-lactamase from *Klebsiella pneumoniae*. Antimicrob Agents Chemother 55:4896–4899. doi:10.1128/AAC.00481-11 21768505 PMC3186949

[71] Elmberg J, Berg C, Lerner H, Waldenstrom J, Hessel R. 2017. Potential disease transmission from wild geese and swans to livestock, poultry and humans: a review of the scientific literature from a One Health perspective. Infect Ecol Epidemiol 7:1300450. doi:10.1080/20008686.2017.1300450 28567210 PMC5443079

[72] Silva G da C, Campana EH, Vasconcelos PC, Silva N da, Santos Filho L, Leite EL, Givisiez PEN, Gebreyes WA, Oliveira C de. 2020. Occurrence of KPC-producing *Escherichia coli* in *Psittaciformes* rescued from trafficking in Paraíba, Brazil. IJERPH 18:95. doi:10.3390/ijerph18010095 33375538 PMC7796378

[73] Dolejska M, Literak I. 2019. Wildlife is overlooked in the epidemiology of medically important antibiotic-resistant bacteria. Antimicrob Agents Chemother 63:e01167-19. doi:10.1128/AAC.01167-19 31209001 PMC6658798

