# Supplementary material for: Wildlife as a reservoir of OXA-48-like carbapenemase-producing Enterobacterales
Source: Microbiol Spectr. 2026 Jul 21;14(9):e00432-26. doi: 10.1128/spectrum.00432-26 (PMC13532257; doi:10.1128/spectrum.00432-26)
Supplement: Supplementary file 1 [file spectrum.00432-26-s0001.docx]

**Wildlife as a reservoir of OXA-48-like carbapenemase producing Enterobacterales**

Imene MEHIDI,^1,2,3^Alima SAIT-GHAROUT,^1^ Abdelaziz Franck BOUGAHAM,^4^ Hocine GOUGAM,^4^ Bogdan I. IORGA,^5^ Riadh MOULAI,^6^ Laurent Dortet, ^2,3,7^ Rémy A. BONNIN,^2,3,7^ Delphine GIRLICH,^2,3^  Mourad AHMIM,^4^ Saoussen OUESLATI,^2,3^ Thierry NAAS^2,3,7^

**Supplementary tables (3) and figures (2)**

**Table S1**. Numbers and distributions of samples from the wildlife in Algeria

| Wild animals | | Bejaia region | | Sétif region | | Jijel region | | Total per sample | |
| --- | --- | --- | --- | --- | --- | --- | --- | --- | --- |
|  |  | # of samples | # of OXA-E | # of samples | # of OXA-E | # of samples | # of OXA-E | # of samples | # of OXA-E |
| Birds | White Wagtail (*Motacilla cinerea*) |  |  | 15 |  | 36 | 1 | 51 | 1 |
|  | Dipper (*Cinclus cinclus*) | 8 |  | 33 |  | 33 | 1 | 74 | 1 |
|  | brown gull (*Lesser Black-backed Gull*) | 18 |  |  |  |  |  | 18 | 0 |
|  | Yellow-legged Gull (*Larus michahellis*) | 101 | 5 |  |  |  |  | 101 | 5 |
|  | black-headed gull (*Chroicocephalus ridibundus*) | 112 | 7 |  |  |  |  | 112 | 7 |
|  | Cattle Egret (*Bubulcus ibis*) | 21 | 2 |  |  |  |  | 21 | 2 |
|  | House swallow (*Delichon urbica*) | 48 | 1 |  |  |  |  | 48 | 1 |
|  | White stork (*Ciconia ciconia*) | 12 |  |  |  |  |  | 12 | 0 |
|  | bats (*Rhinolophus hipposideros*/*Myotis emarginatu*s/*Pipistrellus kuhlii*/*Pipistrellus pipistrellus*) | 515 |  |  |  |  |  | 515 | 0 |
| Land mammals | magot monkey (*Macaca sylvanus*) | 69 | 1 |  |  |  |  | 69 | 1 |
|  | Boar (*Sus scrofa*) | 56 |  |  |  |  |  | 56 | 0 |
| Marine animals | Sea urchins (*Paracentrotus lividus*) | 300 |  |  |  |  |  | 300 | 0 |
|  | Sea bream (*Sparus aurata*) | 31 |  |  |  |  |  | 31 | 0 |
|  | Sea bass (*Paracentrotus lividus*) | 13 |  |  |  |  |  | 13 | 0 |
|  | Saurel (*Trachurus trachurus*) | 100 |  |  |  |  |  | 100 | 0 |
| **Total** | | **1404** | **16** | **48** | **0** | **69** | **2** | **1521** | **18** |

**Table S2.** Numbers and distributions of samples in the captive wildlife in Algeria

| Zoological parks  (# as on fig1, name, city) | | 1  Arche de Noe Oran | | 2  El Hamma  Algiers | | 3  Keffous  Bejaia | | 4  Hadj Aissa  Setif | | 5  El A. Boudjeblida  Jijel | | 6  Brabtia  El kala | |
| --- | --- | --- | --- | --- | --- | --- | --- | --- | --- | --- | --- | --- | --- |
| Niches | | # of samples | # of OXA-48-E | # of sampl | # of OXA-48-E | # of samples | # of OXA-48-E | # of samples | # of OXA-48-E | # of samples | # of OXA-48-E | # of samples | # of OXA-48-E |
| Birds | Fischer's lovebird (*Agapornis fischeri*) |  |  | 2 |  |  |  |  |  |  |  |  |  |
|  | Chukar partridge (*Alectoris Chukar*) |  |  | 2 |  |  |  |  |  |  |  |  |  |
|  | Amazon (*Amazona ochrocephala/amazona auropalliata*) |  |  | 3 |  |  |  |  |  |  |  |  |  |
|  | Amazona amazonica (*Amazone aourou*) |  |  |  |  |  |  |  |  |  |  | 1 |  |
|  | Golden eagle (*Aquila chrysaetos*) | 3 |  | 2 |  | 4 |  |  |  |  |  |  |  |
|  | Blue macaw (*Ara ararauna*) |  |  | 4 |  |  |  |  |  |  |  |  |  |
|  | Eagle owl(*Bubo bubo* ) |  |  |  |  |  |  | 1 |  |  |  |  |  |
|  | Buteo rufinus (*Ferocious hawk*) |  |  | 1 |  | 2 |  | 2 |  |  |  |  |  |
|  | Yellow-crested Cockatoo (*Cacatua galerita*) |  |  | 2 | 1 |  |  |  |  |  |  |  |  |
|  | Canary (*Serinus canaria*) | 3 |  |  |  |  |  |  |  |  |  | 3 |  |
|  | Golden pheasant(*Chrysolophus pictus*) |  |  |  |  |  |  |  |  | 3 |  |  |  |
|  | Quail (*Coturnix coturnix*) |  |  |  |  | 2 |  |  |  |  |  |  |  |
|  | Serin (*Crithagra mozambica*) |  |  | 2 |  |  |  |  |  |  |  |  |  |
|  | Mandarin diamond (*Taeniopygia guttata*) |  |  | 2 |  |  |  |  |  |  |  |  |  |
|  | Emu (*Dromiceius novaehollandiae*) | 1 |  | 2 |  |  |  | 4 |  | 2 |  |  |  |
|  | Peregrine falcon (*Falco peregrinus*) |  |  |  |  | 2 |  | 4 |  |  |  |  |  |
|  | Griffon vulture (*Gyps fulvus*) |  |  | 2 |  | 3 |  | 5 |  | 2 |  | 2 |  |
|  | Silver pheasant (*Lophura nycthemera*) |  |  | 2 | 1 |  |  |  |  |  |  |  |  |
|  | Moluccan ori (*Lorreus garrulus*) |  |  | 1 |  |  |  |  |  |  |  |  |  |
|  | Wavy parakeet (*Melopsittacus undulatus*) | 1 |  | 2 |  |  |  |  |  |  |  |  |  |
|  | Black kite (*Milvus migrans*) |  |  | 3 |  |  |  |  |  |  |  | 3 |  |
|  | Egyptian vulture (*Neophron pecnopterus*) | 3 |  | 1 |  | 2 |  |  |  |  |  |  |  |
|  | Callopsite parakeet (*Nymphicus hollandicus*) |  |  | 6 | 1 |  |  |  |  |  |  |  |  |
|  | Padda java (*Padda oryzivora*) |  |  | 2 |  |  |  |  |  |  |  |  |  |
|  | Blue peacock (*Pavo cristatus*) | 1 |  |  |  |  |  |  |  |  |  |  |  |
|  | Alectoris barbara (*Partridge gambra*) |  |  |  |  | 2 |  |  |  |  |  |  |  |
|  | Common pheasant (*Phasianus colchicus* ) | 1 |  | 6 |  |  |  |  |  |  |  |  |  |
|  | Greater flamingo (*Phoenicopterus ruber roseus*) |  |  | 3 |  |  |  |  |  |  |  |  |  |
|  | Senegal Perrot (*Poicephalus senegalus*) |  |  | 1 |  |  |  |  |  |  |  |  |  |
|  | Parakeet (*Psephotus haematonotus/Cyanoramphus novaezelandiae/Melopsittacus undulatus/Aratinga solstitialis* ) |  |  | 2 |  |  |  |  |  |  |  |  |  |
|  | Gabonese grey (*Psittacus erithacus*) |  |  | 3 | 1 |  |  |  |  | 2 |  |  |  |
|  | Amazon (*Amazona ochrocephala*) , Cockatoo (*Cacatua alba*) |  |  | 3 | 1 |  |  |  |  |  |  |  |  |
|  | Turtle dove (*Streptopelia* ) |  |  | 2 |  |  |  |  |  |  |  |  |  |
|  | African ostrich (*Struthio camelus*) | 1 |  |  |  | 4 |  | 1 |  | 1 |  | 2 |  |
|  | Mandarin diament (*Taeniopygia guttata*) |  |  |  |  | 1 |  |  |  |  |  |  |  |
|  | Lorikeet (*Trichoglossus haematodus*) |  |  | 1 |  |  |  |  |  |  |  |  |  |
| Mammals | Nilgaut antelope (*Boselaphus tragomelus*) |  |  |  |  |  |  |  |  | 1 |  |  |  |
|  | Guinea baboon (*Papio papio*) |  |  |  |  |  |  |  |  | 1 |  |  |  |
|  | Baboon (Papio cynocephalus) |  |  |  |  |  |  | 1 |  |  |  | 3 |  |
|  | Capuchin (Cebus capucinus) |  |  |  |  |  |  |  |  | 2 | 1 |  |  |
|  | Red deer (*Dama dama* ) |  |  | 9 | 1 |  |  | 4 |  | 5 |  | 4 |  |
|  | Golden jackal (*Canis aureus*) |  |  | 5 |  | 1 |  |  |  | 2 |  |  |  |
|  | Black goat (*Capra Hircus Mambrica*) | 1 |  |  |  |  |  |  |  |  |  |  |  |
|  | Chimpanzee (*Pan troglodytes*) |  |  | 3 |  |  |  |  |  |  |  |  |  |
|  | Red coati (*Nasua nasua*) |  |  |  |  |  |  |  |  | 2 |  |  |  |
|  | *Spotted hyena* (Crocuta crocuta) | 1 |  |  |  |  |  |  |  |  |  |  |  |
|  | Dromader (*Camelus dromaderius*) |  |  |  |  |  |  | 1 |  |  |  |  |  |
|  | Fennec (*Vulpes zerda*) | 2 |  | 6 |  |  |  | 2 |  | 3 | 1 |  |  |
|  | Ferret (*Mustela putorius furo*) |  |  | 3 |  |  |  |  |  |  |  |  |  |
|  | Cuvier gazelle (*Gazella cuvieri*) |  |  | 6 | 1 | 3 |  |  |  |  |  |  |  |
|  | Gazelle dorcas (*Gazella dorcas*) |  |  |  |  |  |  | 2 |  | 2 |  |  |  |
|  | Gazelle leptoceros (*Gazella leptoceros*) |  |  | 1 |  |  |  |  |  |  |  |  |  |
|  | Genetta genetta (*Common genet*) |  |  |  |  | 1 |  |  |  |  |  |  |  |
|  | Striped hyena  (*Hyaena hyaena*) |  |  | 1 | 1 | 3 |  |  |  | 3 |  |  |  |
|  | Lama guanaco (*Lama guanicoe*) | 1 |  | 5 |  |  |  | 2 |  | 3 |  | 2 |  |
|  | Dwarf pig ( *sus scrofadomesticus*) |  |  |  |  |  |  |  |  |  |  | 3 |  |
|  | African lion (*Panthera leo)* | 5 |  | 2 |  | 9 |  | 1 |  | 5 |  | 5 |  |
|  | White-cheeked mangabey (*Lophocebus albigena*) |  |  |  |  |  |  |  |  | 3 |  |  |  |
|  | Ichneumon mongoose (*Herpestes ichneumon*) |  |  |  |  |  |  | 2 |  |  |  |  |  |
|  | Striped mongoose (*Mungos mungo*) |  |  |  |  | 1 |  |  |  |  |  |  |  |
|  | Cuffed sheep (*Ammontragus lervia*) |  |  | 4 |  |  |  | 4 |  | 6 |  | 5 |  |
|  | Oryx algazelle (*Oryx dammah*) |  |  |  |  |  |  |  |  | 1 |  |  |  |
|  | Bear (*Ursus arctos* ) |  |  | 2 |  |  |  |  |  | 1 |  | 1 |  |
|  | Panthera tigris (*Panthera tigris* ) | 3 |  | 4 |  |  |  | 4 |  |  |  | 1 |  |
|  | Patas (*Erythrocebus patas*) | 2 |  |  |  |  |  |  |  |  |  |  |  |
|  | Red fox (*Vulpes vulpes* ) | 1 |  |  |  |  |  | 2 |  |  |  |  |  |
|  | Elephant (*Loxodonta africana*) |  |  |  |  |  |  |  |  | 1 | 1 |  |  |
|  | Wild boar (*Sus scrofa*) | 1 |  |  |  | 2 |  | 1 |  | 1 |  |  |  |
|  | Grivet monkey (*Chlorocebus cynosuros*) |  |  | 1 |  |  |  |  |  |  |  |  |  |
|  | Magot monkey (*Macaca sylvanus*) |  |  | 1 |  |  |  | 3 |  | 5 |  | 2 |  |
|  | Patas monkey (*Erythrocebus patas*) |  |  | 2 |  |  |  |  |  |  |  |  |  |
|  | Zebra (*Equus zebra*) |  |  |  |  |  |  |  |  | 1 |  |  |  |
| Others animals | Hare (*Lepus timidus*) |  |  |  |  |  |  | 1 |  |  |  |  |  |
|  | Indian porcupine (*Hystrix indica*) | 1 |  |  |  | 3 |  | 3 |  | 2 |  | 3 |  |
|  | Barbary squirrel (*Atlantoxerus getulus*) | 2 |  |  |  |  |  |  |  |  |  |  |  |
|  | King cobra (*Ophiophagus hannah* ) |  |  |  |  |  |  | 1 |  |  |  |  |  |
|  | Nile crocodile (*Crocodilus niloticus*) | 1 |  | 1 |  |  |  | 1 |  |  |  |  |  |
|  | Lemur catta (*Lemur catta*) |  |  |  |  |  |  |  |  |  |  | 2 |  |
| Feed | | 1 |  | 1 |  | 1 |  | 1 |  | 1 |  | 1 |  |
| Water | | 1 |  | 1 |  | 1 |  | 1 |  | 2 |  | 1 |  |
| Human rectal swabs | |  |  | 9 | 1 | 4 |  |  |  |  |  |  |  |
| **Total** | | **37** | **0** | **129** | **9** | **51** | **0** | **54** | **0** | **62** | **3** | **45** | **0** |

**Table S3: Accession numbers from bioproject PRJNA124256**

| Isolate # | Identification | Accession numbers |
| --- | --- | --- |
| O97H1 | *E. hormaechei hoffmannii* | JBMRJO000000000 |
| O97G10 | *E. hormaechei hoffmannii* | JBMRJN000000000 |
| O98A2 | *E. hormaechei hoffmannii* | JBMRJM000000000 |
| O97I8 | *E. hormaechei hoffmannii* | JBMRJL000000000 |
| O98A6 | *E. hormaechei hoffmannii* | JBMRJK000000000 |
| O98A7 | *E. hormaechei hoffmannii* | JBMRJJ000000000 |
| O97I9 | *E. hormaechei hoffmannii* | JBMRJI000000000 |
| O98A8 | *E. hormaechei hoffmannii* | JBMRJH000000000 |
| O97I10 | *E. hormaechei hoffmannii* | JBMRJG000000000 |
| O97J1 | *E. hormaechei hoffmannii* | JBMRJF000000000 |
| O97J2 | *E. hormaechei hoffmannii* | JBMRJE000000000 |
| O97H7 | *E. hormaechei hoffmannii* | JBMRJD000000000 |
| O97I2 | *E. hormaechei hoffmannii* | JBMRJC000000000 |
| O97H4 | *K. pneumoniae* | JBMRJB000000000 |
| O97J10 | *K. pneumoniae* | JBMRJA000000000 |
| 098A1 | *K. pneumoniae* | JBMRIZ000000000 |
| O98A4 | *K. pneumoniae* | JBMRIY000000000 |
| O97H5 | *K. pneumoniae* | JBMRIX000000000 |
| O97I1 | *K. pneumoniae* | JBMRIW000000000 |
| O97J9 | *K. quasipneumoniae quasipneumoniae* | JBMRIV000000000 |
| O97I4 | *K. quasipneumoniae quasipneumoniae* | JBMRIU000000000 |
| O98A5 | *K. michiganensis* | JBMRIT000000000 |
| O97H9 | *E. coli* | JBMRIS000000000 |
| O97H10 | *E. coli* | JBMRIR000000000 |
| O97I5 | *E. coli* | JBMRIQ000000000 |
| O97H6 | *E. coli* | CP187781 |
| O97H8 | *E. coli* | JBMRIP000000000 |
| O100E1 | *E. coli* | JBSPMA000000000 |
| O97J6 | *R. ornithinolytica* | JBMRIO000000000 |
| O97I7 | *C. freundii* | JBMRIN000000000 |

**Figure S1** : Kieser extracted plasmids of the 30 CPEs. Lane 1: *E. hormaechei hoffmannii* O97H1; Lane 2: *E. hormaechei hoffmannii* O97G10; Lane 3: *E. hormaechei hoffmannii* O98A2; Lane 4: *E. hormaechei hoffmannii* O97I8; Lane 5: *E. hormaechei hoffmannii* O98A6*;* Lane 6*: E. hormaechei hoffmannii* O98A7; Lane 7: O97I9; Lane 8: O98A8; Lane 9: O97I10; Lane 10: O97J1; Lane 11: O97J2; Lane 12: O97H7; Lane 13: O97I2; Lane 14*: K. pneumoniae* O97H4*;* Lane 15*: K. pneumoniae* O97J10*;* Lane 16*: K. pneumoniae* O98A1*;* Lane 17*: K. pneumoniae* O98A4*;* Lane 18*: K. pneumoniae* O97H5*;* Lane 19*: K. pneumoniae* O97I1*;* Lane 20*: K. quasipneumoniae quasipneumoniae* O97J9*;* Lane 21*: K. quasipneumoniae quasipneumoniae* O97J9*;* Lane 22*:* *K. michiganensis* O98A5*;* Lane 23*: E. coli* O97H9*;* Lane 24*: E. coli* O97H10*;* Lane 25*: E. coli* O97I5*;* Lane 26*: E. coli* O97H6*;* Lane 27*: E. coli* O97H8*;* Lane 28*: R. ornithinolytica* O97J6*;* Lane 29*: C. freundii* O97I7, Lane 30*: E. coli* O100E1. Lane M: *E. coli* NCTC 50192 with plasmids of ca. 154, 66, 48, and 7 kb.

**Figure S2:** Kieser extracted plasmids of parental and *E. coli* TOP10 electro-transformant (T). Lane 1 and T1: *E. hormaechei hoffmannii* O97H1; Lane 2 and T2: *E. hormaechei hoffmannii* O98A2; Lane 3 and T3: *E. hormaechei hoffmannii* O97I8; Lane 4 and T4: *E. hormaechei hoffmannii* O98A6*;* Lane 5 and T5*: K. pneumoniae* O97J10*;* Lane 6 and T6*: K. pneumoniae* O98A4*;* Lane 7 and T7*: K. pneumoniae* O97H5*;* Lane 8 and T8*: K. pneumoniae* O97I1*;* Lane 9 and T9*: K. quasipneumoniae quasipneumoniae* O97J9*;* Lane 10 and T10*:* *K. michiganensis* O98A5*;* Lane 11 and T11*: R. ornithinolytica* O97J6*;* Lane 12 and T12*: C. freundii* O97I7; Lane 13 and T13*: E. coli* O100E1*. M: E. coli* NCTC 50192 with plasmids of ca. 154, 66, 48, and 7 kb.
